# A Novel Rrm3 Function in Restricting DNA Replication via an Orc5-Binding Domain Is Genetically Separable from Rrm3 Function as an ATPase/Helicase in Facilitating Fork Progression

**DOI:** 10.1371/journal.pgen.1006451

**Published:** 2016-12-06

**Authors:** Salahuddin Syed, Claus Desler, Lene J. Rasmussen, Kristina H. Schmidt

**Affiliations:** 1 Department of Cell Biology, Microbiology, and Molecular Biology, University of South Florida, Tampa, Florida, United States of America; 2 Graduate Program in Cellular and Molecular Biology, University of South Florida, Tampa, Florida, United States of America; 3 Center for Healthy Aging, Department of Cellular and Molecular Medicine, University of Copenhagen, Copenhagen, Denmark; 4 Cancer Biology and Evolution Program, H. Lee Moffitt Cancer Center and Research Institute, Tampa, Florida, United States of America; The University of North Carolina at Chapel Hill, UNITED STATES

## Abstract

In response to replication stress cells activate the intra-S checkpoint, induce DNA repair pathways, increase nucleotide levels, and inhibit origin firing. Here, we report that Rrm3 associates with a subset of replication origins and controls DNA synthesis during replication stress. The N-terminal domain required for control of DNA synthesis maps to residues 186–212 that are also critical for binding Orc5 of the origin recognition complex. Deletion of this domain is lethal to cells lacking the replication checkpoint mediator Mrc1 and leads to mutations upon exposure to the replication stressor hydroxyurea. This novel Rrm3 function is independent of its established role as an ATPase/helicase in facilitating replication fork progression through polymerase blocking obstacles. Using quantitative mass spectrometry and genetic analyses, we find that the homologous recombination factor Rdh54 and Rad5-dependent error-free DNA damage bypass act as independent mechanisms on DNA lesions that arise when Rrm3 catalytic activity is disrupted whereas these mechanisms are dispensable for DNA damage tolerance when the replication function is disrupted, indicating that the DNA lesions generated by the loss of each Rrm3 function are distinct. Although both lesion types activate the DNA-damage checkpoint, we find that the resultant increase in nucleotide levels is not sufficient for continued DNA synthesis under replication stress. Together, our findings suggest a role of Rrm3, via its Orc5-binding domain, in restricting DNA synthesis that is genetically and physically separable from its established catalytic role in facilitating fork progression through replication blocks.

## Author Summary

When cells duplicate their genome, the replication machinery is constantly at risk of encountering obstacles, including unusual DNA structures, bound proteins, or transcribing polymerases and transcripts. Cells possess DNA helicases that facilitate movement of the replication fork through such obstacles. Here, we report the discovery that one of these DNA helicases, Rrm3, is also required for restricting DNA synthesis under replication stress. We find that the site in Rrm3 critical for this new replication function is also required for binding a subunit of the replication origin recognition complex, which raises the possibility that Rrm3 controls replication by affecting initiation. This is supported by our finding that Rrm3 associates with a subset of replication origins. Rrm3’s ability to restrict replication does not require its helicase activity or the phosphorylation site that regulates this activity. Notably, cells need error-free bypass pathways and homologous recombination to deal with DNA lesions that arise when the helicase function of Rrm3 is disrupted, but not when its replication function is disrupted. This indicates that the DNA lesions that form in the absence of the two distinct Rrm3 function are different, although both activate the DNA-damage checkpoint and are toxic to cells that lack the mediator of the replication checkpoint Mrc1.

## Introduction

The replication machinery is constantly at risk of encountering obstacles such as protein-DNA complexes, DNA secondary structures, transcribing RNA polymerases, RNA-DNA hybrids, and DNA damage, all of which can block fork progression. If these structures cannot immediately be resolved the paused fork may eventually collapse as replisome components become irretrievably inactivated.

The 5’ to 3’ DNA helicase Rrm3 is a member of the Pif1 family, which is conserved from yeast to humans [1,2]. *Saccharomyces cerevisiae RRM3* was first discovered as a suppressor of recombination between tandem arrays and ribosomal DNA (rDNA) repeats [3]. Without Rrm3, extrachromosomal rDNA circles accumulate, suggesting a role in maintaining rDNA repeat stability, and cells accumulate recombination intermediates at stalled replication forks, which has lead to the suggestion that Rrm3 facilitates DNA unwinding and the removal of protein blocks to help fork convergence during replication termination [4–7]. Additionally, replication fork pausing has been observed in the absence of Rrm3 at centromeres, telomeres, tRNA genes, the mating type loci, inactive origins of replication, and RNA polymerase II-transcribed genes [3,5,6].

The mechanism by which Rrm3 aids fork progression is poorly understood, but it is thought that the ATPase/helicase activity of Rrm3 facilitates replication through protein blocks and may also be able to remove RNA transcripts [5,8]. Within each rRNA coding region are two intergenic spacers that contain termination sites that are bound by the replication terminator protein Fob1 to promote fork arrest and to prevent unscheduled transcription [9–11]. Termination site function also requires the intra-S phase checkpoint proteins Tof1 and Csm3, which form a complex with the replisome and antagonize Rrm3 function [12,13]. It is thought that Rrm3 removes Fob1 and other non-histone proteins from DNA before the replication fork encounters them. This ability of Rrm3 to promote replication fork progression is dependent on its catalytic activity [7]. Further supporting a role of Rrm3 in fork progression are synthetic fitness defects or lethality between *rrm3Δ* and mutations that disrupt genes involved in maintaining the integrity of stalled forks, including *rad53Δ*, *mec1Δ*, *srs2Δ*, *sgs1Δ*, *mrc1Δ*, and *rtt101Δ* [5,14–16].

Rrm3 possesses an N-terminal PCNA-interacting peptide (PIP) box, associates with the replication fork *in vivo* and is hyperphosphorylated by Rad53 under replication stress [1,17,18]. The replication damage that arises in the absence of Rrm3 causes constitutive, Mec3/Mec1/Rad9-dependent activation of the checkpoint kinase Rad53 [5,17,19,20]. As a result, Dun1 kinase is activated, leading to degradation of the ribonucleotide reductase (RNR) inhibitor Sml1 and an increase in the dNTP pool [21,22]. This increased dNTP pool has been associated with enhanced DNA synthesis in hydroxyurea (HU) in chromosome instability mutants [22].

Here we show that cells lacking Rrm3 fail to inhibit DNA replication in the presence of HU-induced replication stress and that this failure is not caused by the increased dNTP pool resulting from constitutive DNA-damage checkpoint activation. This novel replication function of Rrm3 is independent of its ATPase/helicase activity and, thus, distinct from Rrm3’s established catalytic role in facilitating fork progression through replication blocks. Instead, we have identified dependency on a novel functional domain in the Rrm3 N-terminus that we find is involved in binding the Orc5 subunit of the origin recognition complex (ORC). Together with our finding that Rrm3 associates with a subset of replication origins, this suggests that Rrm3 may control DNA synthesis by controlling origin activity. Quantitative mass spectrometry and genetic analyses further implicate Rad5-dependent error-free DNA damage bypass and Rdh54 translocase as novel repair mechanisms for DNA lesions that result from inactivating the catalytic activity of Rrm3, whereas these DNA repair factors are dispensable when the Orc5-binding domain is disrupted, leading us to conclude that the types of DNA lesions that result from the inactivation of the two independent Rrm3 functions are distinct.

## Results

### SILAC-based proteomics to identify the cellular response to replication fork pausing

**Fig 1:**
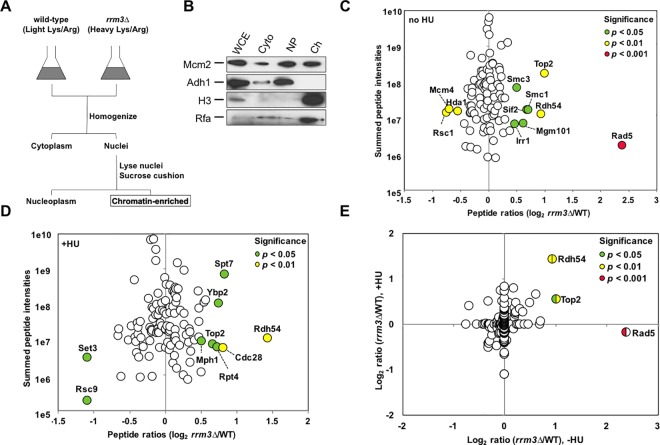
SILAC-based quantification of changes in chromatin association in cells lacking Rrm3. (A) Equal numbers of light-labeled (wildtype) and heavy-labeled (*rrm3Δ*) cells were mixed, nuclei isolated, and the chromatin fraction extracted and analyzed by tandem mass spectrometry using a hybrid linear ion trap-Orbitrap instrument. (B) Subcellular fractionation was verified by following the distribution of proteins in cytoplasmic (Cyto), nucleoplasm (NP), and chromatin (Ch) fractions during the enrichment procedure by Western blotting. Successful chromatin fractionation is indicated by the enrichment of histone H3 and Rfa and the absence of Adh1. Chromatin fractions were analyzed from three biological replicates in (C) the absence of hydroxyurea and (D) in the presence of hydroxyurea. (E) Merger of peptide quantification in the absence and presence of hydroxyurea.

In the absence of Rrm3 cells accumulate replication pause sites at the rDNA locus, in tRNA genes and at centromeric regions, as well as many other sites throughout the genome [5,6,15]. To identify DNA metabolic pathways that deal with stalled forks, we sought to identify proteins whose association with chromatin changed in the absence of Rrm3 using stable isotope labeling by amino acids in cell culture (SILAC)-based quantitative mass spectrometry [23,24]. We extracted the chromatin fraction from nuclei purified from a mixture of wildtype and *rrm3Δ* cells grown in the presence of heavy or light isotopes of arginine and lysine, respectively (Fig 1A and 1B). Across chromatin fractions from three biological replicates we identified 490 peptides from 137 different proteins, with the abundance of 11 proteins changing significantly in at least two of the three replicates (Fig 1C). The largest change in chromatin association was a 5.1-fold increase (*p*<0.001) of Rad5, which belongs to the SWI/SNF family of ATPases and defines an error-free pathway for bypassing replication-blocking DNA lesions [25–28]. The increase in Rad5 was followed by smaller, but significant, increases for Top2 (1.9-fold, *p*<0.01), a type II topoisomerase that is important for the decatenation of replication intermediates, and Rdh54 (1.8-fold, *p*<0.01), a chromatin remodeler with a role in homologous recombination that is still largely unclear. Like Rad5, Rdh54 is a member of the SWI/SNF family of ATPases; it possesses translocase activity on double-stranded (ds) DNA and has been shown to be capable of modifying DNA topology, especially in chromatinized DNA [29–31]. We observed significant decreases in chromatin association for the Rsc1 subunit of the chromatin-structure-remodeling (RSC) complex (2-fold, *p*<0.01), the Mcm4 subunit of the minichromosome maintenance (MCM) replicative DNA helicase (1.9-fold, *p*<0.01), and the catalytic subunit Hda1 of the histone deacetylase (HDAC) complex (1.7-fold, *p*<0.01).

Upon treatment with HU, which induces replication stress by reducing the nucleotide pool [21], Rdh54 abundance in the chromatin fraction of the *rrm3Δ* mutant increased the most (2.6-fold, *p*<0.01) whereas the histone deacetylase Set3 and the Rsc9 subunit of the RSC chromatin remodeling complex saw the largest decreases (2.7-fold, *p*<0.05) (Fig 1D and 1E). The complete list of proteins that underwent significant changes in the HU-treated or untreated *rrm3Δ* mutant, including the FANCM-related Mph1 helicase, the recombination factor Mgm101, and the cohesin components Smc1, Smc3 and Scc3, is provided in S1 Table.

### Rad5 and Rdh54 independently act on DNA lesions that arise in the absence of Rrm3

**Fig 2:**
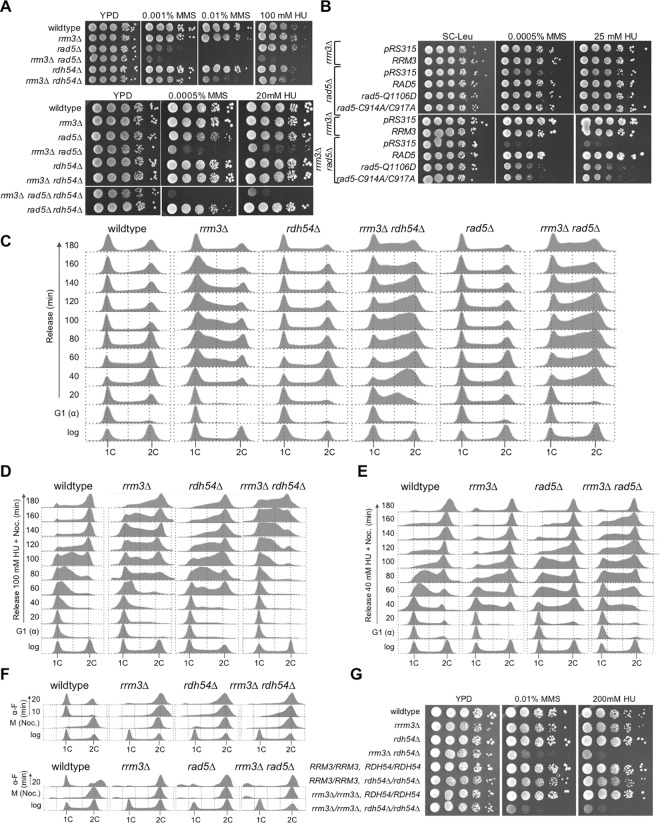
Rad5 and Rdh54 contribute independently to DNA lesion bypass/repair in cells lacking Rrm3. (A) Synergistic increases in DNA-damage sensitivity of *rrm3Δ* mutant lacking *RAD5* and/or *RDH54* were identified by spotting serial dilutions of exponentially growing cultures of the indicated mutants on MMS and HU. (B) Ubiquitin ligase and helicase activities of Rad5 contribute to DNA damage tolerance in the absence of Rrm3. Ubiquitin ligase activity of Rad5 was inactivated by C914A/C917A mutations and ATPase/helicase activity by Q1106D [27,38]. (C) Accumulation of *rrm3Δ* cells in S phase increases upon deletion of *RAD5* or *RDH54*. Cells were synchronized in G1 phase with α-factor, released in YPD to resume the cell cycle, and DNA content analyzed by fluorescence-activated cell sorting every 20 minutes for 3 hours. (D-E) Deletion of *RAD5* or *RDH54* slows progression of *rrm3Δ* cells through S phase in the presence of HU. Cells were synchronized in G1 with α-factor, released into media containing 100 mM HU (*rdh54Δ*) or 40 mM HU (*rad5Δ*) and nocodazole (to trap cells in G2/M). DNA content was analyzed by fluorescence activated cell sorting every 20 minutes for 3 hours. (F) *rrm3Δ*, *rad5Δ*, and *rdh54Δ* mutations slow progression through mitosis into G1 phase. Cells were trapped in mitosis with nocodazole and released into media with α-factor. (G) Deletion of *RRM3* increases DNA damage sensitivity of the haploid and diploid *rdh54Δ* mutant.

Rrm3 helicase is required to prevent excessive replication fork pausing at nonhistone-protein-bound sites, possibly by acting as a protein displacement helicase [6]. The role of Rdh54 as a dsDNA translocase that can act on chromatinized DNA [32,33] and the fork reversal activity of Rad5 suggest that they are recruited to chromatin to recover forks that are blocked due to the lack of Rrm3 or to substitute for Rrm3 in preventing fork pausing. We therefore examined the effect of deleting *RAD5* and *RDH54* in the *rrm3Δ* mutant on genome stability and sensitivity to DNA damage caused by methyl methanesulfonate (MMS) and to replication stress caused by HU. We found synergistic increases in sensitivity to HU and MMS in the *rrm3Δ rad5Δ* and *rrm3Δ rdh54Δ* mutants (Fig 2A). The negative genetic interaction between *rrm3Δ* and *rad5Δ* was particularly strong; both single mutants were no more sensitive to HU than wildtype, but the double mutant failed to form colonies on 100 mM HU and grew very poorly even on 20 mM HU. In contrast to HU, the *rad5Δ* mutant was extremely sensitive to MMS, and deleting *RRM3* led to a further synergistic increase in MMS sensitivity. Inactivation of the ATPase activity of Rrm3 (*rrm3-K260A/D*) caused the same hypersensitivity in the *rad5Δ* mutant as an *RRM3* deletion (Fig 2B). We also identified a negative genetic interaction between *rrm3Δ* and *rdh54Δ*, which was especially strong on MMS. The increased sensitivity of *rdh54Δ* cells to HU and MMS upon deletion of *RRM3* extended to diploid cells (Fig 2G), suggesting that the lesions generated in the absence of Rrm3 are also substrates for recombination between homologous chromosomes that is controlled by Rdh54. Even though the *rrm3Δ rad5Δ* mutant was hypersensitive to MMS and HU, deletion of *RDH54* caused further synergistic increases in sensitivity to both chemicals, indicating that Rad5 and Rdh54 define important pathways for dealing with DNA lesions that arise in the absence of Rrm3, and that they perform (at least some) independent roles. In addition to structure-specific helicase activity, Rad5 also possesses a RING motif associated with ubiquitin ligase activity that plays a role in polyubiquitination of PCNA [34–37]. Two mutations in Rad5, Q1106D and C914A/C917A, were recently described to disrupt its helicase and ubiquitin-ligase activity, respectively [27,38]. Disrupting either of these Rad5 activities in the *rrm3Δ* mutant significantly increased sensitivity to MMS and to HU, indicating that both activities make important contributions to the repair of DNA lesions that arise in the absence of Rrm3 (Fig 2B).

Although Rad5 and Rdh54 chromatin association increased most in the absence of Rrrm3 (Fig 1E), gross-chromosomal rearrangements (GCRs) did not accumulate at higher rates in the *rrm3Δ rad5Δ* or *rrm3Δ rdh54Δ* mutants compared to the single mutants, even after exposure to HU and MMS (Table 1). However, disruption of both, *RAD5* and *RDH54*, in the *rrm3Δ* mutant caused a significant, albeit small, increase in chromosome instability compared to disruption of the single genes, especially upon exposure to HU or MMS, supporting independent contributions of Rdh54 and Rad5-mediated repair mechanisms to genome stability and DNA damage tolerance in the absence of Rrm3. That the effect on the GCR accumulation was small despite a synergistic effect on hypersensitivity to HU and MMS (Fig 2A) could indicate that the DNA lesions are not substrates for GCRs or that chromosomal rearrangements that form in these mutants are not viable.

**Table 1:** Effect of Deletions of *RRM3*, *RAD5*, *RDH54* on Accumulation of Gross-Chromosomal Rearrangements in the Presence or Absence of Genotoxic Agents

Relevant Genotype	GCR Rate					
	Can^r^ 5-FOA^r^ x 10^−10^ (95% CI)					
	Untreated		+ HU		+ MMS	
wildtype	1.1	(<1–6.1)	20	(9.6–35)	65	(55–75)
*rrm3Δ*	14	(11–27)	139	(106–171)	97	(90–114)
*rad5Δ*	237	(220–271)	1477	(1300–1590)	928	(837–1020)
*rrm3Δ rad5Δ*	260	(224–263)	1600	(1390–1680)	1040	(834–1220)
*rdh54Δ*	17	(11–27)	191	(144–212)	108	(87–135)
*rrm3Δ rdh54Δ*	25	(14–54)	202	(194–224)	120	(99–266)
*rad5Δ rdh54Δ*	263	(244–272)	1578	(1320–1710)	991	(907–1160)
*rrm3Δ rad5Δ rdh54Δ*	322	(278–419)	2446	(2140–2670)	1664	(1350–1810)

Whereas *rdh54Δ* and *rad5Δ* cells moved through an undisturbed cell cycle with similar kinetics as wildtype cells, *rrm3Δ* cells were delayed in progressing to G2/M, consistent with previous observations [6,14]. We find that this delay was enhanced when *RDH54* or *RAD5* were deleted (Fig 2C). To examine progression of *rrm3Δ rad5Δ* and *rrm3Δ rdh54Δ* cells through S phase under replication stress, we released α-factor arrested cells from G1 phase in the presence of HU and trapped them in G2/M with nocodazole (Fig 2D and 2E). After 140 minutes, virtually all wildtype cells had reached 2C DNA content, whereas *rrm3Δ* and *rdh54Δ* showed a marked delay (Fig 2D, 120 minute time point). When we combined *rrm3Δ* and *rdh54Δ* mutations, this slowdown was so severe that most cells still had near 1C DNA content 100 minutes after release from G1 arrest, consistent with the synergistic increase in HU hypersensitivity of the *rrm3Δ rdh54Δ* mutant. Similarly, *rad5Δ rrm3Δ* cells were delayed in reaching 2C DNA content in HU (Fig 2E). However, all mutants were able to recover from a 2-hour arrest in 100 mM HU and resume the cell cycle normally (S1 Fig). When we examined the ability of nocodazole-arrested cells in G2/M to complete mitosis and reach G1 phase, we found that most wildtype cells were in G1 after 10 minutes, whereas *rrm3Δ*, *rad5Δ*, *rdh54* cells and the double mutants showed 2C DNA content 20 minutes after release (Fig 2F). Together, these findings indicate that Rad5 and Rdh54 facilitate the progression of *rrm3Δ* cells through S phase, both in the presence and in the absence of HU, and that in the absence of Rrm3, cells accumulate DNA damage that impairs exit from mitosis.

In addition to Rad5 and Rdh54, which exhibited the most significant increases in chromatin association in the absence of Rrm3 (Fig 1E), we tested DNA damage sensitivity of cells that lacked Rrm3 in combination with other nonessential factors revealed in the proteome screen (Fig 1C and 1D), including Mgm101, Hda1, Set3, and Mph1. Whereas deletions of *MGM101*, *HDA1* or *SET3* had no effect on sensitivity of wildtype or *rrm3Δ* cells to HU or MMS (S2 Fig), deletion of *MPH1* caused a synergistic increase in HU and MMS sensitivity of the *rrm3Δ* mutant (S3 Fig), consistent with our previous finding [39]. In the absence of Mph1, *rrm3Δ* cells progressed very slowly through an undisturbed cell cycle and accumulated in G2/M when they were released from a 2-hour incubation in 100 mM HU (S3B and S3C Fig). When cells were released from HU arrest into media with 40 mM HU and α-factor, virtually all wildtype cells and the single mutants were trapped in G1 phase after 60 minutes (with a slight S phase delay in the *mph1Δ* mutant), whereas the majority of *rrm3Δ mph1Δ* cells accumulated in S phase, never forming a majority peak at 1C DNA content in the 120-minute time course (S3D Fig). Deleting *MPH1* in the *rrm3Δ rad5Δ* mutant led to a slight increase in sensitivity to HU compared to the double mutants, but not to MMS (S3E Fig). These findings implicate Mph1 as another crucial factor for overcoming spontaneous and DNA-damage-induced replication-blocking lesions when Rrm3 is absent and suggest pathways for error-free bypass of DNA polymerase blocking lesions, implicated by Rad5 and Mph1, and homologous recombination, implicated by Rdh54, as independent mechanisms that are recruited to chromatin to act on blocked replication forks.

### A novel function of Rrm3 in controlling DNA replication maps to the N-terminal tail and is independent of Rrm3 catalytic activity

**Fig 3:**
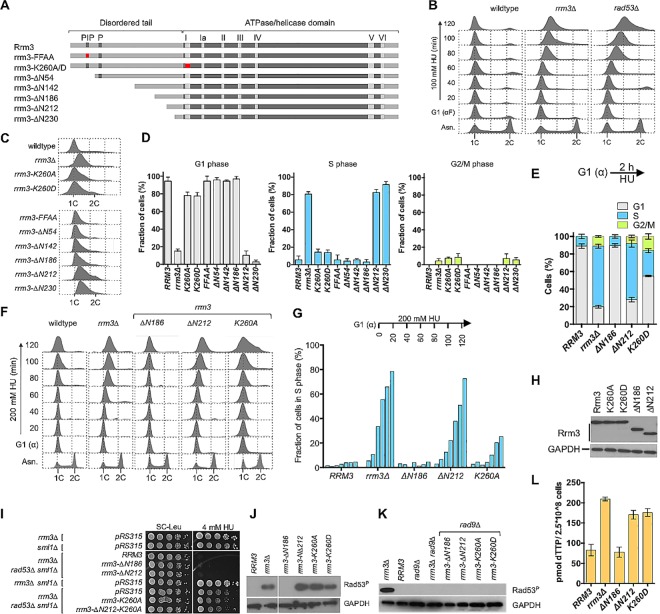
A 26-residue region in the N-terminal tail of Rrm3 is required for the control of DNA replication under replication stress. (A) Rrm3 consist of a ~230-residue disordered N-terminal tail and a ~400 residue ordered C-terminal ATPase/helicase domain. A putative PCNA-interaction motif (PIP) between residues 35–42 [18] and a cluster of phosphorylated residues (P) between residues 85–95 [17] are indicated by gray boxes in the disordered tail. N-terminal tail truncations (*rrm3ΔN54*, *rrm3ΔN142*, *rrm3ΔN186*, *rrm3ΔN212*, *rrm3ΔN230*) and point mutations designed to inactivate the PIP box (*rrm3-FFAA)* and the Walker A motif of the helicase domain (*rrm3-K260A*, *rrm3-K260D*) were constructed. Point mutations are indicated by a red box. (B) The *rrm3Δ* mutant continues DNA replication in the presence of HU, but not in MMS (S4B Fig). Cells were synchronized in G1 phase with α-factor and released into media containing 100 mM HU. DNA content was analyzed by fluorescence-activated cell sorting (FACS) every 20 minutes for 2 hours after G1 release. (C) DNA content analysis by FACS of *rrm3* mutants shows continued DNA replication in *rrm3Δ*, *rrm3-ΔN212* and *rrm3-ΔN230* mutants during a 2-hour incubation in 200 mM hydroxyurea, but maintenance of the peak at 1C DNA content for wildtype cells and the other *rrm3* mutants. (D) Distribution of cells throughout the cell cycle phases from FACS analysis was quantified with FlowJo v.10.1. Experiments were performed in triplicate and the mean with standard deviation is reported. (E) Cell cycle distribution based on cell morphology. (F) Cells expressing an N-terminal truncations of 212 residues continue DNA replication in the presence of 200 mM HU whereas the majority of cells expressing a 186-residue truncation or the ATPase/helicase-dead allele maintain a peak at 1C DNA content like wildtype cells. Cells were synchronized in G1 with α-factor and released into 200 mM HU for 2 hours. Aliquots were removed prior to release from G1 and then every 20 minutes for 2 hours and DNA content analyzed by FACS. (G) Fraction of cells in S phase was quantified every 20 minutes over the 2 hour time course in (F) with FlowJo v.10.1 software. (H) Expression of *rrm3* alleles was analyzed by Western blot using an antibody against a C-terminal myc-epitope. (I) Rrm3 truncated by 212 N-terminal residues is an active ATPase/helicase. Deletion of *RRM3* or inactivation of its ATPase/helicase activity suppresses HU hypersensitivity of the *rad53Δ* mutant [17] whereas the *rrm3-ΔN186* and *rrm3-ΔN212* alleles do not, exhibiting ATPase/helicase activity of the *RRM3* wildtype allele. (J) Rad53 is constitutively activated in *rrm3Δ*, *rrm3-K260A*, *rrm3-K260D* and *rrm3-ΔN212* mutants. Cells were arrested in G1 with α-factor and Rad53 phosphorylation analyzed 30 min after release from arrest with a phospho-specific Rad53 antibody. Rad53 activation in *rrm3* mutants correlates with an increased dNTP pool, but not with continued DNA synthesis in the presence of hydroxyurea. (K) Rad53 phosphorylation in *rrm3* mutants, irrespective of continued DNA replication in HU or catalytic activity, is Rad9-dependent. (L) Nucleotide pool, represented here by dTTP, is increased in *rrm3Δ* cells and in cells expressing the *rrm3-ΔN212* truncation or the ATPase/helicase-dead *rrm3-K260A* mutant, but not in cells expressing the shorter *rrm3-ΔN186* truncation.

All functions of the Rrm3 helicase known to date are dependent on its ATPase/helicase activity. During our analysis of cell cycle progression, however, we observed that cells with a deletion of *RRM3* continue to replicate DNA in the presence of HU, similar to a *rad53Δ* checkpoint mutant, whereas the helicase-defective *rrm3-K260A* and *rrm3-K260D* mutants maintained near 1C DNA content after 2 hours in HU, similar to wildtype (Fig 3B and 3C). This observation suggested the presence of a previously unknown, ATPase/helicase-independent function of Rrm3 in DNA replication. Since this replication defect was independent of the ATPase/helicase activity located in the ordered C-terminal domain of Rrm3 (residues 250–723), we explored a possible involvement of the 230-residue, disordered N-terminal tail (Fig 3A, S4A Fig). The only motifs previously identified in this tail are a putative PCNA-interacting peptide (PIP) box between residues 35–42 [18] and a cluster of phosphorylated residues between S85 and S92 [17]. Deletion or mutation of the PIP-box (*rrm3-ΔN54*, *rrm3-FFAA*) had no effect on DNA replication in HU, whereas deletion of the entire N-terminal tail (*rrm3-ΔN230*) caused the same replication defect as deleting *RRM3* (*rrm3Δ*) (Fig 3C). By constructing a series of N-terminal truncations (Fig 3A and 3C) we determined that a deletion of up to 186 residues, which also eliminates the PIP-box and the phosphorylation site, was able to maintain the wildtype replication phenotype in HU, whereas deletions of 212 or 230 residues caused the same inability to restrict DNA synthesis in HU as *rrm3Δ* (Fig 3D–3G), thus narrowing down the critical functional site for control of DNA replication to the 26 residues between residues 186–212. This defect was not due to changes in protein stability or levels of expression of the *rrm3* mutant alleles (Fig 3H), which had been observed for other Rrm3 truncations before [20]. The importance of residues 186–212 for controlling DNA replication was limited to HU, and not observed when cells were exposed to the alkylating agent MMS (S4B Fig).

Deletion of *RRM3* or inactivation of its ATPase/helicase activity was recently reported to partially suppress the HU hypersensitivity of the *rad53Δ* mutant [17]. We obtained the same findings, and observed that the new *rrm3*-*ΔN212* allele does not act as a suppressor (Fig 3I), indicating that the *rrm3-ΔN212* allele codes for a functional ATPase/helicase. To verify this, we disrupted the Walker A motif in the *rrm3-ΔN212* mutant (*rrm3-Δ212-K260A*) and, as expected, it now suppressed the HU hypersensitivity of the *rad53Δ* mutant to the same extent as the ATPase-defective *rrm3-K260A* allele (Fig 3I).

The Rad53 checkpoint kinase was constitutively activated in the *rrm3-Δ212* mutant just like in the ATPase/helicase-defective *rrm3-K260A/D* mutants, and Rad53 activation in both mutants was dependent on the mediator of the DNA damage checkpoint Rad9 (Fig 3J and 3K). Through degradation of the ribonucleotide reductase (RNR) inhibitor Sml1, the nucleotide pool increases upon Rad53 activation, and this correlates with enhanced fork progression [22]. We found that the *rrm3* mutants that continued DNA replication in HU (*rrm3Δ*, *rrm3ΔN212*) as well as the *rrm3* mutant that maintained a peak at 1C DNA content (*rrm3*-*K260D*) had constitutively increased nucleotide pools upon entrance into S phase (Fig 3L), indicating that the continued DNA replication in HU seen in the *rrm3-ΔN212* mutant could not be explained by a larger nucleotide reservoir prior to its depletion by HU addition. In fact, based on quantification of the cell cycle profiles obtained by flow cytometry and by visual analysis of morphology the vast majority of *rrm3Δ*, *rrm3-ΔN212* and *rrm3-ΔN230* cells entered S phase in HU and continued to progress, whereas the majority of wildtype cells and the other *rrm3* mutants did not enter S phase during the 2-hour incubation in HU, with the peaks of DNA content remaining at 1C (Fig 3B–3G, S4C Fig).

Together, these findings suggest a new function of Rrm3 in restricting DNA replication in the presence of HU and prevention of S phase damage, which maps to residues 186–212 of the N-terminal tail and does not require Rrm3’s established activity as an ATPase/DNA helicase.

### Residues of Rrm3 required for control of DNA replication are critical for Orc5 binding

**Fig 4:**
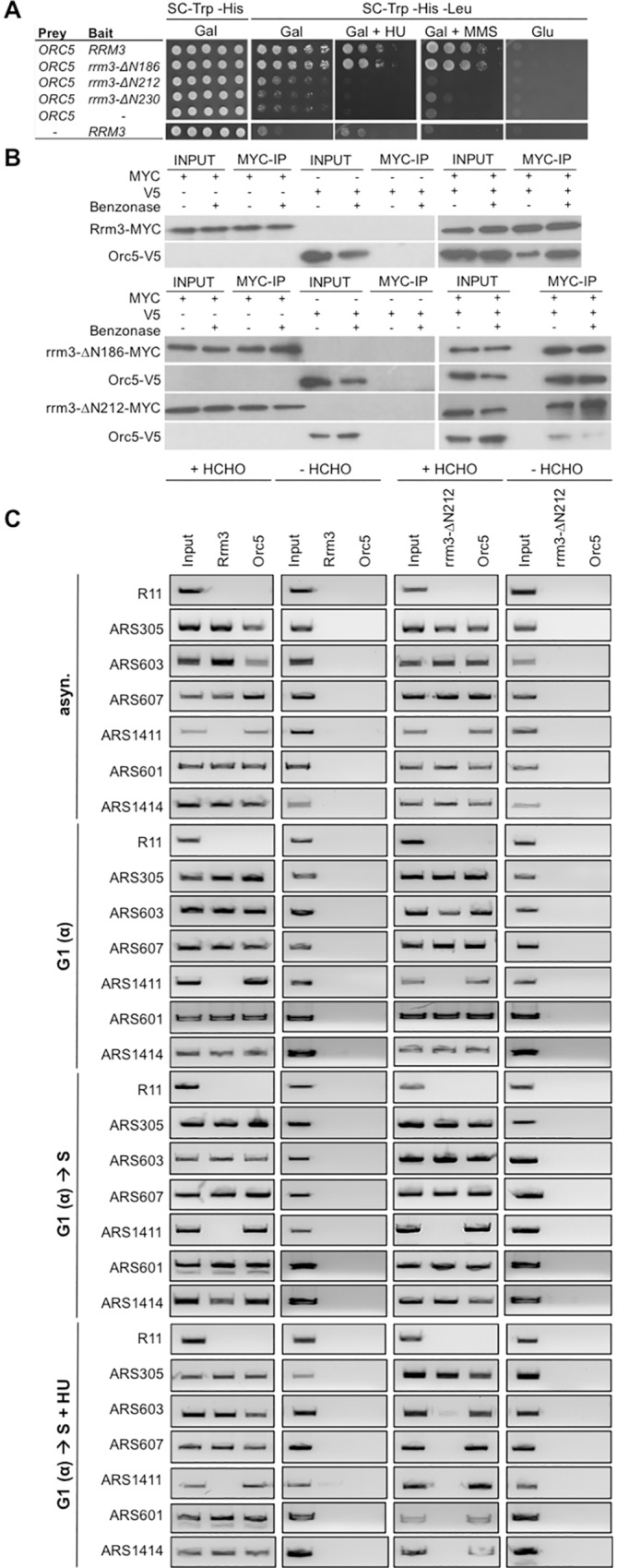
The N-terminal Rrm3 region that controls DNA replication is an Orc5-binding site and is involved in association of Rrm3 with a subset of origins of replication. (A) Residues 186–212 of Rrm3 are required for physical interaction with origin recognition complex (ORC) subunit Orc5. Orc5 binding to wildtype Rrm3 and rrm3 mutants was assessed by yeast-two-hybrid analysis on media lacking leucine to identify cells expressing the *LEU2* reporter gene upon bait-prey binding. Rrm3 truncated by 186 residues binds Orc5, in the presence or absence of HU or MMS, whereas deletion of an additional 26 residues (*rrm3-0ΔN212*) eliminates binding of Orc5. (B) Interaction between Rrm3, rrm3-ΔN186 and rrm3-ΔN212 with Orc5 was analyzed by co-immunoprecipitation in the presence and absence of benzonase. (C) Ability of Rrm3 and rrm3-ΔN212 to associate with origins of replication was analyzed by chromatin-immunoprecipitation in cells from asynchronous cultures (asyn.), cultures synchronized in G1 phase with α-factor (G1(α)), and cultures released from α-factor arrest into S phase for 45 minutes in the absence (G1(α) → S) or presence of 200 mM hydroxyurea (G1(α) → S + HU). Association with 12 early and late origins was investigated (see also S4D Fig) with and without cross-linking with formaldehyde (HCHO). R11 is a non-origin containing control sequence in the *BRR2* gene on chromosome V.

Long disordered tails, such as the N-terminal 230 residues of Rrm3 that extend from a structured catalytic core, typically serve as sites for protein binding and posttranslational modification [40]. The phenotype of the *rrm3-ΔN212* allele in the *rad53Δ* mutant indicates that it encodes a proficient ATPase/helicase, suggesting that the replication defect of this allele is caused by loss of a protein-binding site in the disordered tail. Because deletion of the putative PIP-box and the recently identified phosphorylation site did not impair the ability of Rrm3 to control DNA replication, we explored the possibility that Orc5, an ATP-binding subunit of the origin recognition complex (ORC), binds to the N-terminal tail of Rrm3. An interaction between the two full-length proteins had previously been identified in a yeast-two-hybrid screen [41]. When we combined *ORC5* with the various *rrm3* truncation alleles in a yeast two-hybrid assay, we found that deletion of 186 residues did not diminish Orc5 binding to Rrm3, in the presence or absence of MMS or HU, whereas deletion of 212 or 230 residues eliminated binding (Fig 4A). To verify the importance of the N-terminal region of Rrm3 for Orc5 binding in vivo we tested the ability of Orc5 to co-immunoprecipitate myc-epitope-tagged Rrm3, rrm3-ΔN186 and rrm3-ΔN212. Consistent with the yeast-two hybrid assay, wildtype Rrm3 and rrm3-ΔN186 bound efficiently to Orc5 whereas binding of rrm3-ΔN212 was impaired (Fig 4B). These findings show that the same site of Rrm3 that restricts DNA replication in HU is required for a physical interaction with Orc5 in vivo and raise the possibility that Rrm3 may control DNA replication by affecting replication origins.

### Orc5-binding site of Rrm3 is required for association of Rrm3 with a subset of origins of replication in the presence of HU but not in undisturbed S phase

To test the hypothesis that Rrm3 acts on replication origins we tested if Rrm3 and rrm3-ΔN212 associate with origins and if this association is affected by the presence of HU. Since progression of *rrm3*Δ and *rrm3-ΔN212* mutants into S phase in the presence of HU is not as pronounced as in the absence of the Rad53 checkpoint kinase, which modulates the timing of origin firing and S phase progression upon exposure to HU, we considered that Rrm3 might act only on a subset of origins. We therefore selected a variety of replication origins for analysis by chromatin immunoprecipitation (ChIP), ranging from early to late-initiating origins and including two origins near telomeres (ARS319, ARS501). We performed ChIP on asynchronous cultures, cultures synchronized in G1 with α-factor, as well as cultures that were released from G1 into S phase for 45 minutes in the presence or absence of HU. We observed that Rrm3 and rrm3-ΔN212 associated with ARS305, ARS601, ARS603, ARS607, and ARS1414 in asynchronous cultures, in G1 and in S phase, but not with ARS1411 (Fig 4C). In fact, the lack of a PCR product for ARS1411 in the asynchronous culture indicates that Rrm3 does not associate with this replication origin for any extended period during the cell cycle; Rrm3 was also not at ARS306, ARS319, ARS416, ARS522, ARS606, and ARS609 (S4D Fig). This suggests that Rrm3 associates with a subset of origins of replication in unperturbed G1 and S phase independently of its Orc5-binding site. However, when we released cells into S phase in the presence of 200 mM HU, rrm3-ΔN212 lost its association with ARS602, ARS603, ARS607, and ARS1414 whereas wildtype Rrm3 remained bound (Fig 4C), suggesting that the failure of the rrm3-ΔN212 mutant to halt DNA synthesis and progression into S phase in the presence of HU might be due to a failure of rrm3-ΔN212 to act on a subset of replication origins (40% of origins tested in this study).

### Differential requirements of Rrm3 functions in controlling DNA replication and ATPase/helicase activity in cells lacking DNA repair factors or replication checkpoint components Mrc1 and Tof1

**Fig 5:**
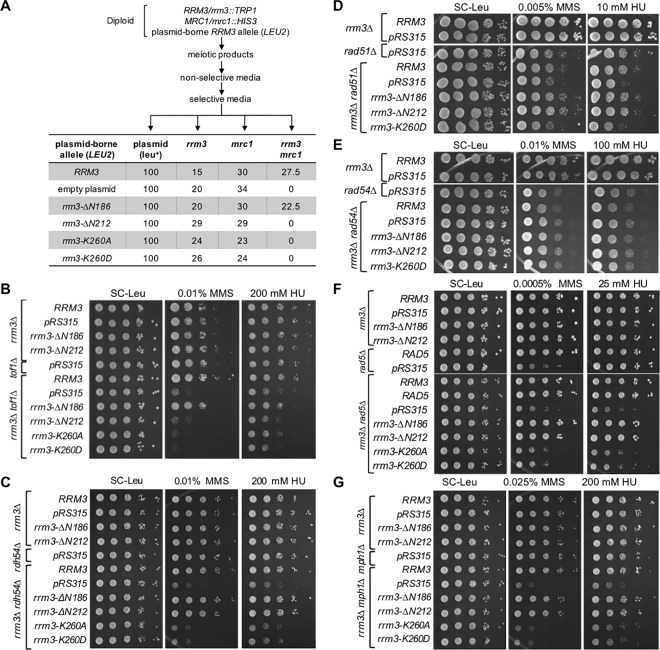
Orc5-binding site of Rrm3 is required for cell viability and tolerance to HU and MMS in the absence of Mrc1/Tof1 but not in the absence of Rad5, Rad51, Rdh54 or Mph1. (A) Residues 186–212 of Rrm3 are required for *mrc1Δ* viability. Diploids heterozygous for *mrc1Δ* and *rrm3Δ* mutations were transformed with plasmids expressing N-terminal truncations of Rrm3 and 100 meiotic products that grew on SC-Leu media, indicating the presence of the plasmid-borne *RRM3* alleles, were genotyped. Absence of meiotic products that grew on SC-Leu-Trp-His indicates synthetic lethality between the *rrm3* allele and *mrc1*Δ. (B) Absence of residues 186–212 of Rrm3 increases sensitivity of the *tof1Δ* mutant to HU and MMS. (C-G) Requirement of the ATPase/helicase activity of Rrm3, but not the Orc5-binding site between residues 186–212, for suppression of HU and MMS hypersensitivity of *rdh54Δ*, *rad51Δ*, *rad5Δ* and *mph1Δ* mutants. *RRM3* is not required in the *rad54Δ* mutant for tolerating HU or MMS.

To investigate the link between Rrm3 functions and DNA replication, we examined the replication checkpoint. Replication mutants exhibit strong genetic interactions with Mrc1/Claspin, which acts as a mediator of the replication stress checkpoint–a Rad9-independent pathway of the intra-S-phase checkpoint [42–46]. Mrc1 is also a component of normal replication forks, which is loaded at origins of replication and stays associated with the replisome [42,44,47,48]. Mrc1, like Rrm3, is required for efficient replication [49]. The function of Mrc1 in DNA replication is essential for the viability of cells lacking Rrm3 [47] whereas Mrc1 phosphorylation on SQ and TQ sites linked to its checkpoint function is dispensable [19]. However, the role of this functional interaction between Rrm3 and Mrc1 in DNA replication has remained unclear. We therefore tested if the ability of Rrm3 to control DNA replication was required for the viability of the *mrc1Δ* mutant. For this purpose, we transformed diploids heterozygous for the *mrc1Δ* and *rrm3Δ* mutations with plasmids expressing N-terminal truncations of Rrm3 and analyzed the viability of meiotic products. Fig 5A shows that the *rrm3*-Δ*N186* allele restored viability to the *rrm3*Δ *mrc1*Δ mutant as effectively as the wildtype *RRM3* allele, whereas the helicase-dead alleles and the *rrm3*-Δ*N212* allele were as ineffective as the null allele (empty plasmid). Thus the helicase activity of Rrm3 is not sufficient for viability of the *mrc1*Δ mutant; Rrm3’s new Orc5-binding domain for controlling DNA replication is also required.

In addition to Mrc1, Tof1 promotes normal progression of the replication fork; however, in contrast to Mrc1, its requirement for fork progression appears more limited, assisting primarily replication through non-histone protein complexes with DNA [50]. *TOF1* deletion was not lethal in the *rrm3Δ* mutant and neither single mutant was hypersensitive to HU or MMS. The combined loss of Rrm3 and Tof1, however, caused a synergistic increase in DNA-damage sensitivity (Fig 5B). Identical to the functional requirements in the absence of Mrc1 both, the ATPase/helicase activity of Rrm3 and the Orc5 binding domain, were required for growth in the presence of DNA damage and replication stress in the absence of Tof1.

In contrast to *mrc1Δ* and *tof1Δ* mutants, we found that only the ATPase/helicase activity of Rrm3 was required for the suppression of HU and MMS hypersensitivity of the *rdh54Δ* mutant (Fig 5C). The N-terminal tail, including its function in controlling DNA replication, was dispensable, with the *rrm3-ΔN212* allele exhibiting a wildtype phenotype in the *rdh54Δ* mutant. When we extended this analysis to the Rdh54 homolog Rad54, and the HR factor Rad51, which both Rdh54 and Rad54 interact with [29,51], we observed that the *rrm3* alleles caused the same phenotypes in the *rad51Δ* mutant as in the *rdh54Δ* mutant, but had no effect on the *rad54Δ* mutant (Fig 5D and 5E). Like *rdh54Δ* and *rad51Δ* mutants, *rad5Δ* and *mph1Δ* mutants exhibited increased sensitivity to HU and MMS only if the ATPase activity of Rrm3 was disrupted, whereas the *rrm3*-*ΔN212* allele caused the same phenotype as the *RRM3* wildtype allele (Fig 5F and 5G).

Together, these findings suggest two separable functions of Rrm3 in DNA replication. First, an ATPase/helicase-dependent function that facilitates fork progression through protein-DNA complexes, which if disrupted (*rrm3-K260A/D*) causes aberrant replication intermediates that can be rescued by Rad5, Rdh54/Rad51 or Mph1 mechanisms. Second, an N-terminal function that restricts DNA replication in the presence of HU, mediated by Rrm3 association with replication origins, which if disrupted (*rrm3-ΔN212*) requires the replication checkpoint factors Mrc1 and Tof1 for viability and DNA damage survival. This differential requirement of factors involved in DNA repair and DNA damage tolerance pathways in the *rrm3-ΔN212* and *rrm3-K260A/D* mutants also suggests that the types of DNA lesions that accumulate upon inactivation of the two Rrm3 functions are different, but both lead to dependence on Mrc1 for survival and both are sufficient for constitutive activation of the DNA-damage checkpoint.

### Requirement of the Orc5-binding domain of Rrm3 for suppression of HU-induced mutations, but not MMS-induced mutations and gross-chromosomal rearrangements

If Rrm3 is important for the response to replication stress induced by HU, cells lacking the catalytic activity of Rrm3 or its Orc5-binding domain might be prone to accumulating mutations at higher rates than wildtype cells. To test this, we measured forward mutation rates at the *CAN1* locus and the accumulation of GCRs on chromosome V in the presence and absence of HU or MMS (Table 2).

**Table 2:** Accumulation of Forward Mutations at *CAN1* and Gross-chromosomal Rearrangements in Untreated *rrm3* Mutants and after Treatment with Genotoxic Agents

Relevant genotype	*CAN1* forward mutation rate			GCR rate		
	x 10^−7^ (95% CI)			x 10^−10^ (95% CI)		
	Untreated	+HU	+MMS	Untreated	+HU	+MMS
wildtype	1.6 (1.4–1.9)	2.3 (2.0–2.8)	19 (17–23)	1.1 (<1–6.1)	20 (9.6–35)	65 (55–75)
*rrm3Δ*	5.1 (4.3–5.8)	8.1 (7.4–8.8)	32 (28–34)	14 (11–27)	139 (106–171)	97 (90–114)
*rrm3-ΔN186*	1.9 (1.7–2.3)	2.5 (2.2–2.9)	23 (19–24)	4.4 (<1–8.8)	43 (18–47)	56 (39–71)
*rrm3-ΔN212*	2.8 (2.6–3.0)	4.1 (4.0–4.5)	21 (20–24)	4.5 (<1–13)	33 (17–39)	78 (63–92)
*rrm3-K260A*	4.3 (4.0–5.0)	8.0 (7.9–8.4)	30 (27–31)	14 (10–23)	158 (105–201)	93 (81–117)
*rrm3-K260D*	4.4 (4.3–4.8)	8.2 (7.6–9.2)	29 (27–33)	18 (10–25)	132 (110–170)	97 (88–113)

Two-fold (*ung1Δ*) to 50-fold (*rad27Δ*) increases in *CAN1* forward mutation rates compared to wildtype have previously been reported for numerous DNA metabolism mutants [52]. Deletion of *RRM3* or disruption of its ATPase/helicase activity caused a significant increase in spontaneous *CAN1* mutations (Table 2). Of the truncation alleles, which encode catalytically active *rrm3* mutants (Fig 3I), *rrm3Δ*-*N186* was indistinguishable from wildtype whereas *rrm3Δ*-*N212* caused a small, but significant, increase in the *CAN1* mutation rate in untreated cells and upon exposure to HU. In contrast, expression of the *rrm3Δ*-*N212* allele had no effect on the *CAN1* mutation rate if cells were treated with MMS, consistent with our observation that the *rrm3-ΔN212* mutant exhibits a defect in controlling replication in HU, but not MMS. GCRs accumulated at increased rates in the *rrm3Δ* and *rrm3-K260A/D* mutants in the absence and presence of HU or MMS, but accumulated at wildtype levels in cells expressing N-terminal truncations under all conditions. These mutator phenotypes, albeit mild, reveal that Rrm3’s ATPase/helicase activity helps to suppress all tested mutation types induced by either HU or MMS, or in their absence, whereas the N-terminal plays a role specifically in the suppression of spontaneous and HU-induced mutations, but not for the suppression of MMS-induced mutations, or GCRs under any conditions.

## Discussion

By quantifying changes in chromatin composition we have identified Rad5 and Rdh54 as novel factors that respond to increased replication fork stalling induced by the absence of Rrm3, and affirmed the importance of Mph1. These factors suggest that error-free post-replicative repair (PRR), implicated by Rad5 and Mph1, and HR, implicated by Rdh54, act on DNA polymerase blocking sites that arise throughout the genome in the absence of Rrm3. The N-terminal unstructured tail, including the Orc5-binding site identified in this study, is dispensable for this ATPase/helicase-dependent role of Rrm3 in facilitating fork progression. Instead, we have discovered that the N-terminal tail encodes a new function of Rrm3 –to control DNA replication in the presence of HU. This function of Rrm3 is distinct from its established role as an ATPase/helicase, is not regulated by the previously identified phosphorylation cluster [17] or the PIP-box [18] and, in contrast to the ATPase/helicase activity of Rrm3, does not contribute to the HU hypersensitivity of the *rad53Δ* mutant.

Based on changes in DNA content as measured by flow cytometry, we observed that wildtype cells maintained near 1C DNA content for 180 minutes after release from G1 phase into HU, whereas *rad53Δ*, *rrm3Δ* and *rrm3*-*ΔN212* did so for only 60 minutes (Fig 3, S4C Fig). The extent of continuing DNA replication in the presence of HU, however, was not as pronounced in the *rrm3* mutants as in the *rad53Δ* mutant. Although the DNA-damage checkpoint is chronically activated in the *rrm3*-*ΔN212* mutant and, as a consequence, nucleotide levels are increased as cells are about to enter S phase, these increased nucleotide levels do not appear to be not sufficient for ability of the *rrm3-ΔN212* mutant to continue DNA replication upon HU exposure because the *rrm3*-*K260A/D* mutants showed the same nucleotide level increase and DNA-damage checkpoint activation, but maintained a peak at 1C DNA content in HU with only a small percentage of cells entering S phase (Fig 3G).

**Fig 6:**
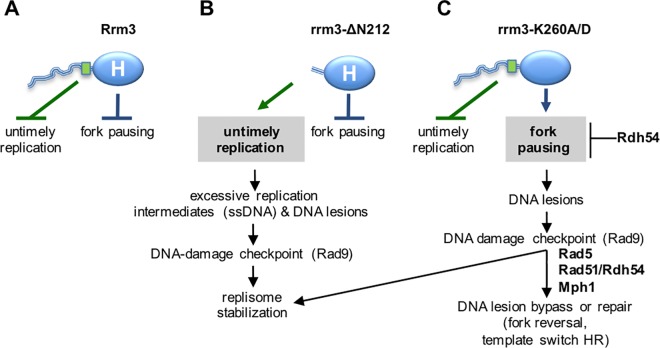
Rrm3 performs two genetically and physically separable functions during DNA replication. (A) Rrm3 controls DNA synthesis via residues 186–212 (green rectangle) in its disordered N-terminal tail (blue line). Rrm3 associates with a subset of early and late-initiating origins of replication in G1 and S phase. Through binding Orc5 and maintaining association of Rrm3 with origins during replication stress, we propose that the N-terminal tail of Rrm3 may control DNA synthesis by controlling some origin activity to prevent untimely replication during replication stress. The N-terminal tail may also play a role in unperturbed cells as indicated by constitutive checkpoint activation in its absence. Independently of its N-terminal tail, Rrm3 associates with the replisome and utilizes its ATPase/helicase activity (blue oval labeled H) to facilitate fork progression through replication blocks. (B) Deleting the N-terminal tail of Rrm3 disrupts Orc5 binding and association with origins and causes enhanced DNA synthesis and S phase progression in HU, as well as accumulation of point mutations in normal S-phase and in HU. Excessive accumulation of replication intermediates and ensuing DNA lesions are the most probable cause of DNA-damage checkpoint activation in these cells. Despite loss of the N-terminal function, the ATPase/helicase-dependent function of Rrm3 in fork progression through blockages is intact, suggesting that Rrm3 can be recruited to replisomes independently of the N-terminal tail. (C) Disrupting the ATPase/helicase activity of Rrm3 maintains control over DNA synthesis, but forks are unable to progress through replication blocks, leading to DNA lesions that require Rad5, Rad51, Rdh54, and Mph1 for bypass or repair and activate the DNA damage checkpoint. In contrast to loss of the Orc5-binding site, a small increase in GCRs is observed in addition to point mutations, indicating the formation of different types of DNA lesions in the ATPase/helicase mutant, most likely DNA breaks.

Therefore, considering Rrm3’s known function as an accessory ATPase/helicase that facilitates progression of the replication fork, and its new function in controlling DNA synthesis reported here, we propose a model (Fig 6) where Rrm3 performs two genetically and physically separable functions to deal with challenges during genome duplication: (1) the N-terminal tail of Rrm3 plays a structural role in preventing untimely replication in the presence of replication stress (HU) and in normal S phase, and (2) the C-terminal ATPase/helicase domain plays a catalytic role in preventing fork pausing. The site between residues 186 to 212, which is in a segment of the N-terminal tail not previously assigned a function, is not only involved in restricting DNA synthesis in HU, but also for the association of Rrm3 with Orc5 and a subset of origins of replication. That rrm3-ΔN212 fails to restrict DNA synthesis and S phase progression in the presence of HU suggests that the association of Rrm3 with origins of replication is inhibitory and that this inhibition is realized through binding Orc5, the ATP-binding subunit of ORC. By binding Orc5, Rrm3 could act as an inhibitor of ORC ATPase activity, which is required for loading of minichromosome maintenance (MCM) proteins and for initiation of DNA replication [53,54], or Rrm3 could block the recruitment of another replication protein. Although ORC is associated with origins throughout the cell cycle, Orc5 does not appear to play a role in the completion of S phase, or the remainder of the cell cycle [55,56].

Besides the association of Rrm3 with origins in HU, which depends on the N-terminal tail, our ChIP data from the rrm3-ΔN212 mutant also show association with origins in normal G1 and in unperturbed S phase in a manner that does not require the N-terminal tail, invoking the presence of another protein binding site in the rrm3-ΔN212 polypeptide, which is made up almost entirely of the catalytic domain. Indeed, the phenotype of *rrm3-ΔN212* mutants deficient in HR (*rdh54Δ*, *rad51Δ*) or PRR (*rad5Δ*, *mph1Δ*) suggests that the association of Rrm3 with the replisome [1], appears to occur outside of the N-terminal tail. While the N-terminal tail is required for binding Orc5, it is not required for association with origins of replication, unless cells are exposed to replication stress. Together with continued DNA synthesis and progression into S phase in the presence of HU our findings, thus, raise the possibility that Rrm3 performs a replication checkpoint-like function in response to HU.

Instead of a global role in controlling origin activity, the wildtype level of HU sensitivity of *rrm3Δ* cells, the less pronounced S phase progression in HU than that of the *rad53*Δ mutant, and the importance of Rrm3 for replicating through certain nonhistone-protein-bound regions suggest that Rrm3 may play a role at origins in specific loci, such as those in highly transcribed regions and regions with converging transcription, which are often late-firing [57], rRNA and tRNA coding loci, or highly transcribed metabolic genes, where ORC has been found to be bound to the open reading frames, possibly to coordinate the timing of replication with transcription [58]. Indeed, our analysis so far has revealed that Rrm3 appears to associate only with a subset of replication origins. The group of origins not associated with Rrm3 includes early and late replicating origins as well as two origins near telomeres. It is currently unclear what distinguishes the origins that associate with Rrm3 from those that do not. For example, there are no consistent differences in how their activity in HU is affected by mutations in the DNA-damage checkpoint or the DNA replication checkpoint [59], the time they are activated [60,61], or any obvious chromosomal features. Association with some but not other origins in unperturbed G1 and S phase does indicate, however, that Rrm3 interacts with a factor specific to some origins rather than a replication protein common to all pre-RCs or replisomes.

The role of Rrm3 at certain origins in HU is likely to be the cause of enhanced DNA synthesis and S phase progression in the *rrm3Δ* and *rrm3-ΔN212* mutants. However, DNA-damage checkpoint activation in *rrm3-ΔN212*, but not *rrm3-ΔN186*, and synthetic lethality between *mrc1*Δ and *rrm3*-ΔN212, but not *rrm3-*Δ*N186*, suggest that there are conditions of replication stress other than exposure to HU that require the integrity of the Orc5-binding domain of Rrm3.

The role of Rrm3 in controlling DNA replication is not affected by inactivation of the ATPase/helicase activity (Fig 6C). Instead, it impairs Rrm3’s established function in facilitating fork progression through replication blocks, leading to the accumulation of DNA lesions that activate the DNA-damage checkpoint and can give rise to mutations. By identifying changes in chromatin composition combined with genetic assays we have identified Rad5 and Rdh54 as novel factors that contribute to the maintenance of genome stability in the absence of Rrm3’s ATPase/helicase activity. Rad5 defines an error-free pathway for the bypass of DNA polymerase blocking lesions [26–28,62–64]. As a structure-specific DNA helicase, Rad5 is capable of regressing replication forks *in vitro* [25]. Such a regressed fork is thought to provide an alternative template for DNA synthesis, generating enough nascent DNA to eventually bypass the replication block. The ATPase activity of Rad5 and the RING motif involved in polyubiquitination of PCNA [34–36,65] contribute to DNA damage tolerance in the absence of Rrm3. Evidence for a role of the ATPase activity of Rad5 in remodeling blocked replication forks has been obtained *in vitro* [25] whereas a role of Rad5-dependent polyubiquitination of PCNA in activating HR-dependent template switching has more recently been suggested [66]. Evidence that these two Rad5 activities can function independently, as we determined here in the *rrm3Δ* mutant, was also observed for bypass of MMS-induced lesions by sister-chromatid recombination [66].

Besides fork regression, Rad5 has also been implicated in DNA damage bypass by HR-dependent template switching between sister-chromatids [66] and the major HR factors Rad51, Rad52 and Rad54 as well as Sgs1 have been implicated in error-free DNA lesion bypass [67]. It was therefore surprising that Rdh54, a dsDNA translocase that is known to play a major role in meiotic, but not mitotic, HR [68–70], is recruited to chromatin when Rrm3 is absent—both in the presence and absence of HU. Rdh54 was only required in the absence of the ATPase/helicase activity of Rrm3, but not in the absence of the Orc5-binding domain, implicating Rdh54 in repair of DNA lesions that arise when Rrm3 cannot facilitate fork progression through replication blocks. Even though Rdh54 does not affect gene conversion repair of a DSB, a role specifically in repair that involves template switches was recently reported [71], and could be related to its increased chromatin association and the DNA-damage hypersensitivity of the *rrm3Δ rdh54Δ* and *rrm3-K260A rdh54Δ* mutants. That Rad51 was required for this repair, but Rad54 was not, suggests a Rad51-dependent HR pathway in mitotic cells where Rdh54 takes the place of Rad54.

Although it is unknown how Rdh54 acts in template switching, its activities *in vitro* seem compatible with those that may be required to rescue a paused fork. Like Rad5 and the human Rad5 ortholog, HTLF, Rdh54 is a dsDNA translocase of the SWI/SNF family [29,30,72]. *In vitro*, it can dislodge Rad51 from dsDNA and introduces negative supercoiling into dsDNA that can cause strand separation [29–31]. These Rdh54 activities could help to regulate restart at fork pause sites in Rad5-mediated pathways, such as fork regression/reversal or template switching, and in HR-mediated events. Whereas Rdh54 can remove proteins from dsDNA and remodel chromatinized DNA, an ability to remove bound proteins from DNA has not yet been shown for Rad5, and RecQ-like helicases are only capable of acting on forked DNA structures that are protein-free [32,33]. The synergistic interactions between *rad5Δ* and *rdh54Δ* in the absence of *RRM3* clearly identify a requirement of Rdh54 outside of a Rad5 mechanism. In addition to facilitating template switching HR when error-free PRR is inactivated, Rdh54 could act in the avoidance of replication fork pausing in a manner similar to Rrm3 by removing certain proteins from dsDNA, such as shown for Rad51, which appears to have a tendency to associate with nonrecombinogenic dsDNA [29,31,70].

That the ATPase activity of Rrm3 is required in the absence of Rad5, Rdh54, Rad51 or Mph1, whereas the role of Rrm3 in controlling DNA replication is dispensable strongly suggests that the types of DNA damage checkpoint activating DNA lesions in the *rrm3-K260A* and *rrm3-ΔN212* mutants are different, and that Rad5, Rdh54, Rad51 and Mph1 act on DNA lesions that form when replications forks are unable to move through obstacles, but not on DNA lesions that form during untimely DNA replication (*rrm3-ΔN212* mutant). In contrast, Mrc1 and Tof1 were required for viability and DNA damage tolerance when either of the two Rrm3 activities was disrupted. Mrc1, the mediator of the replication stress checkpoint, mediates Rad53 phosphorylation specifically in response to replication fork pausing, leading to intra-S checkpoint activation and inhibition of late-origin firing [44,59]. That synthetic lethality between *rrm3Δ* and *mrc1* is limited to those *mrc1* alleles that cause DNA damage accumulation during S phase [49], whereas the checkpoint function of Mrc1 is dispensable [19,49] suggests that the additive accumulation of S phase damage due to lack of both, Mrc1 and Rrm3, is lethal and suggests that dysregulated replication in the *rrm3-ΔN212* mutant (Fig 6B) also leads to S phase damage, consistent with our observation of Rad9-dependent activation of Rad53.

Finally, the new N-terminal Rrm3 function in controlling DNA replication is separated from Rrm3’s established C-terminal function as an ATPase/helicase in facilitating fork progression not only by the differential requirement for Rad5, Rdh54, Rad51 and Mph1, but also by different spontaneous and DNA-damage induced mutation spectra. This supports that the N-terminal tail is neither involved in the recruitment of Rrm3 to active replication forks nor in facilitating fork progression through protein-bound sites, and that a separate replisome binding site is likely to be located in the ATPase/helicase domain. The accumulation of GCRs and point mutations in the ATPase /helicase mutant, spontaneously or induced by HU or MMS, could be indicative of DNA break formation as a result of replication fork stalling. In contrast, the Orc5-binding domain mutant did not accumulate GCRs under any conditions, suggesting wildtype levels of DNA breaks, including in HU and MMS, but increasingly formed point mutations. That these point mutations formed specifically in response to HU, but not MMS, suggests that they arise during the enhanced DNA synthesis that occurs in this mutant in HU.

In summary, this study has revealed a 26-residue region in Rrm3 that is critical for a novel, ATPase-independent function of Rrm3 in preventing untimely DNA replication and for binding Orc5, which appear to be mechanistically linked. We also identified association of Rrm3 with a subset of replication origins and the dependence of this association on the N-terminal tail under replication stress, but not in unperturbed cells. Genome-wide ChIP and quantification of DNA synthesis in cells expressing the new *rrm3* alleles will help to reveal the regions undergoing untimely DNA replication and provide further insight into the mechanism at replication origins underlying continued DNA synthesis under replication stress and lethality with *mrc1Δ*. That yeast has two DNA helicases (Rrm3, Pif1) that belong to the Pif1 family, whereas multicellular eukaryotes where the Pif 1 helicase family is conserved [2] typically have one (e.g. Pif1 in humans), might be an indication that Rrm3’s role in DNA replication is highly specialized to control replication and facilitate fork progression in genomic regions that are distinctively organized in yeast and to deal with the high gene density imposed on its small genome that requires tight coordination between replication initiation and ongoing transcription.

## Materials and methods

### Stable isotope labeling by amino acids in cell culture (SILAC) and isolation of chromatin fraction

For double isotope labeling of lysine and arginine, yeast strain KHSY5144 (*lys2Δ arg4Δ*) was grown at 30°C with and vigorous shaking for at least ten generations in “heavy” medium (6.9 g/l yeast nitrogen base without amino acids [Formedium], 1.85 g/l amino acid dropout mixture without arginine and lysine [Kaiser formulation, Formedium], 2% glucose, 15 mg/l [^13^C_6_] L-arginine and 30 mg/l [^13^C_6_] or [^13^C_6_, ^15^N_2_] L-lysine). KHSY5143 (*lys2Δ arg4Δ rrm3Δ*) was grown in “light” medium, containing 15 mg/l L-arginine and 30 mg/l L-lysine at 30°C with and vigorous shaking.

Chromatin was isolated using a method adapted from [73]. Approximately 4 x 10^9^ cells were resuspended in 10 ml of 100 mM PIPES/KOH, pH 9.4, 10 mM dithio- treitol (DTT), 0.1% sodium azide, then incubated for 10 min at room temperature, followed by incubation in 10 ml of 50 mM KH_2_PO_4_/K_2_HPO_4_, pH 7.4, 0.6 M sorbitol, 10 mM DTT, containing 200 mg/ml Zymolyase-100T and 5% Glusulase at 37°C for 30 min with occasional mixing. Spheroplasts were washed with 5 ml of ice-cold wash buffer (20 mM KH_2_PO_4_/K_2_HPO_4_, pH 6.5, 0.6 M sorbitol, 1 mM MgCl_2_, 1 mM DTT, 20 mM beta-glycerophosphate, 1 mM phenyl-methylsulfonyl fluoride (PMSF), Protease inhibitor tablets (EDTA free, Roche) and resuspended in 5 ml of ice-cold wash buffer. The suspension was overlaid onto 5 ml of 7.5% Ficoll-Sorbitol cushion buffer (7.5% Ficoll, 20 mM KH_2_PO_4_/K_2_HPO_4_, pH 6.5, 0.6 M sorbitol, 1 mM MgCl2, 1 mM DTT, 20 mM beta-glycerophosphate, 1 mM PMSF, Protease inhibitor tablets) and spheroplasts were spun through the cushion buffer at 5000 rpm for 5 min to remove proteases derived from Zymolyase-100T. Pelleted spheroplasts were resuspended in 200 ml of ice-cold wash buffer and dropped into 18% Ficoll, 20 mM KH_2_PO_4_/K_2_HPO_4_, pH 6.5, 1 mM MgCl_2_, 1 mM DTT, 20 mM beta-glycerophosphate, 1 mM PMSF, Protease inhibitor tablets, 0.01% Nonidet P-40, with stirring. After homogenization, unbroken cells were removed by two spins (5000 x *g* for 5 min at 4°C). Nuclei were pelleted by centrifugation at 16,100 x *g* for 20 min and the cytoplasmic fraction removed. After washing in ice-cold wash buffer, nuclei were resuspended in 200 ml of EB buffer (50 mM HEPES/KOH, pH 7.5, 100 mM KCl, 2.5 mM MgCl_2_, 0.1 mM ZnSO_4_, 2 mM NaF, 0.5 mM spermidine, 1 mM DTT, 20 mM beta-glycerophosphate, 1 mM PMSF, protease inhibitor tablets) and lysed by addition of Triton X-100 to 0.25%, followed by incubation on ice for 10 min. Lysate was overlaid on 500 ml of EB buffer, 30% sucrose, 0.25% Triton X-100, and spun at 12,000 rpm for 10 min at 4°C. The top layer was removed and the chromatin pellet was washed in 1 ml of EB buffer, 0.25% Triton X-100 and spun at 10,000 rpm for 2 min at 4°C.

### Sample preparation and LC-MS/MS

The chromatin pellet was resuspended in 40 μl of 1.5x Tris-Glycine SDS sample buffer and incubated for 2 min at 85°C. DTT was added to a final concentration of 5 mM and incubated for 25 min at 56°C. Iodoacetamide was added to 14 mM final concentration and incubated for 30 min at room temperature in the dark. DTT was added to a final concentration of 5 mM and incubated for 15 minutes at room temperature in the dark. The protein mixture was diluted 1:5 in 25 mM Tris-HCl, pH 8.2; CaCl_2_ was added at a final concentration of 1 mM, and trypsin was added at 4–5 ng/μl, followed by incubation at 37°C overnight. Trifluoroacetic acid was added to 0.4% final concentration and centrifuged at 2,500 x *g* for 10 min at room temperature. Peptides in the supernatant were desalted using reverse-phase tC18 SepPak solid-phase extraction cartridges (Waters). The sample was eluted with 5 ml of 50% acetonitrile and lyophilized. The lyophilized product was resuspended in 0.1% formic acid prior to tandem mass spectrometric analysis on an LTQ Orbitrap XL (Thermo). Scans on the Orbitrap were obtained at a mass resolving power of 60000 at *m/z* 400 and top 10 abundant ions were selected for fragmentation in the LTQ ion trap. Further processing of the RAW files was done in MaxQuant version 1.5.3.30 [74] against the Saccharomyces genome database (SGD). A database of known contaminants in MaxQuant was used as well as constant modification of cysteine by carbamidomethylation and variable modification of methionine oxidation. The first search tolerance was set at 20 ppm, then 8 ppm tolerance for the main search. Fragment ion mass tolerance was set to 0.5 Da and the minimum peptide length was 6 amino acids. Unique and razor peptides were used for identification and the false discovery rate was set to 1% for peptides and proteins [74,75]. Statistical analysis of the data was carried out with Perseus software using an approach by Benjamini and Hochberg [76].

### Yeast strains and plasmids

Yeast strains used in this study are derived from S288C background. Strains for SILAC labeling and chromatin fractionation were derived from KHSY5036 (*MAT ɑ*, *ura3-52*, *trp1Δ63*, *his3Δ200*). Yeast strains used in DNA-damage sensitivity and mutation assays were derived from KHSY802 (*MAT ɑ*, *ura3-52*, *trp1Δ63*, *his3Δ200*, *leu2Δ1*, *lys2Bgl*, *hom3-10*, *ade2Δ1*, *ade8*, *hxt13*::*URA3*). Gene deletions were carried out as described using HR-mediated integration of a selectable cassette [77]. Mutants containing more than one gene deletion or mutation were obtained by random spore isolation from diploids heterozygous for the desired mutations. Point mutations were introduced in plasmids by site-directed mutagenesis (QuickChange, Stratagene) and verified by sequencing. *RRM3* truncations were created in plasmid pKHS137 and plasmid pJG4-5* using HR-mediated integration in KHSY2331 (*lig4Δ*) and verified by sequencing. Yeast was grown at 30°C in yeast extract (10g/l), peptone (20g/l), dextrose (20g/l), media (YPD) with or without Bacto agar, or in synthetic complete (SC) media (yeast nitrogen base 6.7g/l, dextrose 20g/l) supplemented with the appropriate amino acid mix. All yeast strains and plasmids used in this study are listed in S2 and S3 Tables, respectively.

### Fluctuation assays

Gross-chromosomal rearrangement (GCR) rates were determined by fluctuation analysis by taking the median rate of at least 15 cultures from at least two isolates and are shown with 95% confidence intervals [78–80]. Cells with GCRs were identified by their resistance to canavanine and 5-fluoro-orotic acid (Can^r^ 5-FOA^r^), which is indicative of simultaneous inactivation of *CAN1* and *URA3* on chromosome V, on selective media as previously described [80]. To observe GCRs after exposure to DNA-damaging agents HU and MMS, cells were grown to an OD_600_ = 0.5, released into medium containing the drug and were cultured for 2 hours at 30^°^C. Cells were then released into fresh YPD medium and grown for 24 hours before being plated. Forward mutation rates were determined by fluctuation analysis by method of the median as previously described and are shown with 95% confidence intervals [78,81,82]. Briefly, fifteen cultures from at least two different isolates were grown overnight at 30^°^C in 3 ml of YPD media. Dilutions were plated on YPD agar to determine the viable cell count, and 500 μl was plated on synthetic media supplemented with 60 μg/ml canavanine, but lacking arginine to select for *can1* mutants. To obtain forward mutation rates and GCR rates after exposure to MMS and HU, cells were grown to OD_600_ = 0.5, released into medium containing the drug and cultured for 2 hours at 30^°^C. Cells were then released into fresh YPD medium and grown for 24 hours before being subjected to fluctuation analysis.

### DNA damage sensitivity assay

Cell cultures were grown as indicated either in YPD or selective media to maintain plasmids (SC-Leu) to OD_600_ = 0.5, and 10-fold serial dilutions were spotted on YPD or SC-Leu supplemented with methyl methanesulfonate (Sigma Aldrich) or hydroxyurea (US Biological) at the indicated concentrations. Colony growth was recorded after 2 to 3 days of incubation at 30^°^C.

### DNA content analysis

Cells were prepared for DNA content analysis as previously described [83]. Briefly, cells were washed and fixed in 70% ethanol for 1 hour at room temperature, sonicated in 50 mM sodium citrate (pH 7), washed in 50 mM sodium citrate (pH7), and RNAse A was added to a final concentration of 250 μg/ml. After overnight incubation at 37^°^C, cells were washed in 50 mM sodium citrate and stained with Sytox green (Molecular Probes) at a final concentration of 1 μM in the dark at room temperature for 1 hour immediately prior to fluorescence-activated cell sorting (FACS) on a BLD LSR II analyzer. The distribution of cells throughout the cell cycle phases was quantified with the FlowJo v8.3.3 software.

### Western blot analysis

Cells were grown to OD_600_ = 0.5 in YPD at 30^°^C, synchronized in G_1_ with α-factor (15 μg/ml), released into fresh, pre-warmed YPD. Cells were harvested after 30 min, immediately put on ice and adjusted for cell number. Whole cell extracts were prepared with 20% trichloroacetic acid as previously described [84] and separated by 10% SDS-PAGE for Western blot analysis. Phospho-specific Rad53 antibody EL7 was a gift from A. Pellicioli (FIRC Institute of Molecular Oncology Foundation, Milan, Italy). Antibody sc-6680 (SCBT) was used for Mcm2, ab34680 (Abcam) for Adh1, ab46765 (Abcam) for histone H3, AS07214 (Agrisera) for Rfa1, and MMS-150P (Covance) for the myc-epitope.

### Co-immunoprecipitation

Cells expressing Orc5-V5-6xHis and either Rrm3-myc, rrm3-ΔN186-myc, or rrm3-ΔN212-myc were grown to an OD_600_ ~ 1.0 and cells were harvested by centrifugation. Cells were re-suspended in cell lysis buffer (50 mM HEPES pH 7.5, 10% v/v glycerol, 140 mM NaCl, 1 mM EDTA, 0.5% Igepal, 1 mM PMSF, EDTA-free Protease inhibitors (Pierce)) and vortexed in a cell disruptor with glass beads for 5 minutes. Cell lysates were cleared by centrifugation at 4°C and lysates were split in half. 1 mM PMSF and 20 mM MgCl_2_ was added to all samples, and 300 U of Benzonase (Sigma) was added to one half of the sample whereas the other half was not treated. Cell lysates were placed on ice for 30 minutes, added magnetic beads coated with to C-MYC antibody and incubated with mixing for 2 h at 4°C. Beads were washed thoroughly 10 times in 1 ml of cell lysis buffer, re-suspended in protein sample buffer, and boiled for 5 min. Samples were separated on a 7% SDS-PAGE gel and presence of Rrm3.myc, rrm3-ΔN186.myc, rrm3-ΔN212.myc, Orc5.V5.6xHIS and GAPDH was determined by western blotting using monoclonal antibodies against myc (Covance), V5 (Sigma), and GAPDH (Pierce).

### Chromatin-immunoprecipitation

Fifty millilters of cells corresponding to 2.0 x 10^7^ cells/ml were incubated for 15 min at room temperature with or without 1% formaldehyde and harvested. Cell pellets were washed twice with PBS, re-suspended in 600 μl of cell lysis buffer (50 mM HEPES pH 7.5, 140 mM NaCl, 1 mM EDTA, 1% Triton X-100, 0.1% sodium deoxycholate, 1 mM PMSF, 1 mM benzamidine, 1 μg/ml aprotinin, 1 μg/ml leupeptine, and 1 ug/ml pepstatin), lysed in a cell disruptor with glass beads at 4°C, and sonicated four times for 20 s each at 4°C. The lysate was clarified by centrifugation and whole cell extract was added to magnetic beads coated with c-myc antibody or V5 antibody, followed by incubation for 60 min at 4°C. Beads were washed four times in cell lysis buffer, three times in TE buffer (10 mM Tris-Cl, pH 7.5, 1 mM EDTA), and re-suspended in TE/1% SDS buffer. Sample was incubated at 65°C for 10 min and processed for DNA purification as previously described [85]. Sequences of primers used for PCR are available upon request.

### Determination of dNTP levels

Cells grown to stationary phase were transferred to acidic media (pH 3.5) and grown to logarithmic phase. Cells were synchronized in G1 phase over two hours with the addition of 2 μg/ml alpha-factor (Genscript) every hour. Cells were washed twice with sterile water. 2.5×10^8^ yeast cells were pelleted, resuspended in 1 ml 60% methanol, and disrupted by 5 consecutive freeze and thaw cycles using liquid nitrogen and warm water, followed by incubation at -20°C for 90 minutes and boiling at 100°C for 3 minutes. The lysate was centrifuged at 19000×g for 15 minutes and the supernatant frozen in liquid nitrogen. Methanol was evaporated in a SpeedVac (Thermo Scientific) and the residue was rehydrated in 100 μl Ultra-pure H_2_O (Invitrogen, GIBCO). Determination of cellular dNTP concentration was performed as earlier described [86]. Each extraction was performed at least in triplicate.

### Yeast Two-hybrid Assay

Yeast strain EGY48 containing reporter plasmid pSH18-34 was co-transformed with a lexA-fusion bait vector (pEG202) and a B42-tagged prey vector (pJG4-5^*^), and transformants were selected on synthetic complete (SC) medium plates lacking histidine and tryptophan (SC-Trp-His). Single transformants were purified and resuspended in liquid SC-His-Trp medium containing 2% galactose and 1% raffinose, and grown overnight at 30°C. Cultures were diluted to OD_600_ = 0.2, grown to OD_600_ = 0.8, and 2-fold serial dilutions were spotted onto SC-His-Trp-Leu containing either 2% glucose or 2% galactose with or without hydroxyurea (HU) or methyl methanesulfonate (MMS). Colony growth was recorded after 72 hours.

### Analysis of meiotic products

Diploids heterozygous for the desired mutant alleles (*rrm3*::*TRP1*, *mrc1*::*HIS3*) transformed with plasmid-borne alleles of *RRM3* (linked to *LEU2*) were sporulated by nitrogen starvation in 0.1% potassium acetate for 5 days at 30°C with vigorous shaking. Asci were incubated in the presence of 500 μg/ml of zymolase in 1M sorbitol for 20 min at 30°C, enriched for meiotic products as previously described [87] and plated on nonselective media (YPD). After incubation for 2 days at 30°C, colonies were spotted on SC-Leu media and 100 leu^+^ colonies genotyped further by spotting on SC-Leu-Trp, SC-Leu-His, and SC-Leu-Trp-His to identify *rrm3*Δ, *mrc1*Δ and *rrm3*Δ *mrc1*Δ mutants, respectively, all harboring various plasmid borne *RRM3* alleles.

### Morphological analysis of yeast cells

Cells were grown to early log phase and incubated with 100 mM hydroxyurea for 2 hours at 30°C with shaking. Cells were fixed with 3.7% formaldehyde, permeablized with ethanol, and mounted in Vectashield medium with DAPI (Vectorlabs). Cells were examined using an EVOS fluorescence microscope and grouped into unbudded (G1 phase), small-budded (S phase, bud is up to one-fourth of the diameter of mother cell), or large-budded (G2/M, bud is equal to or greater than one-fourth of the diameter of the mother cell). 200 cells for each yeast strain were scored.

## Supporting Information

S1 Fig Rad5 and Rdh54 are not required for normal S phase progression of *rrm3Δ* cells after release from 2 hour exposure to 100 mM HU. (TIF)

S2 Fig Deletion of the nonessential *HDA1*, *SET3*, or *MGM101* genes does not affect the DNA damage sensitivity of the *rrm3Δ* mutant. Chromatin association of Hda1 and Set3 significantly decreased in the absence of Rrm3 whereas Mgm101 increased. Deletion of *MGM101* resulted in the ‘petite’ phenotype. Serial dilutions of exponentially growing cultures of the indicated mutants were spotted on rich media containing 0.01% MMS or 100 mM HU, or no drug (YPD), followed by incubation for 2–3 days at 30℃.
(TIF)

S3 Fig Requirement of Mph1 in the absence of Rrm3. (A) Deletion of *RRM3* causes a synergistic increase in HU and MMS sensitivity of cells lacking the DNA helicase Mph1. Absence of Mph1 causes delayed S phase progression of *rrm3Δ* cells in the absence of HU (B), after release from HU into an undisturbed S phase (C), and, most severely, during chronic exposure to HU (D). (E) Adding the *mph1*Δ mutation to the *rrm3*Δ *rad5*Δ mutations leads to a slight increase in sensitivity to 50 mM HU, but not to 0.0005% MMS.
(TIF)

S4 Fig Effect of Rrm3 on DNA replication. (A) The N-terminal tail of Rrm3 is predicted to be unstructured. A disorder score of >0.5 indicates a disordered residue [88]. (B) In contrast to HU, *rrm3-ΔN212 and rrm3Δ* mutants arrest with 1C DNA content when exposed to MMS. (C) DNA content analysis of an *rrm3Δ* mutant released from G1 arrest into 100 mM HU. (D) Rrm3 and rrm3-ΔN212 do not associate with ARS306, ARS319, ARS416, ARS501, ARS606 and ARS609. Association with origins of replication was analyzed by chromatin-immunoprecipitation in cells from asynchronous cultures with or without cross-linking with formaldehyde (HCHO).
(TIF)

S1 Table Proteins in the chromatin fraction that undergo significant changes in cells lacking Rrm3 (PDF)

S2 Table Yeast strains used in this study (PDF)

S3 Table Plasmids used in this study (PDF)
