# Supplementary figures and images for: A Novel Rrm3 Function in Restricting DNA Replication via an Orc5-Binding Domain Is Genetically Separable from Rrm3 Function as an ATPase/Helicase in Facilitating Fork Progression
Source: PLoS Genet. 2016 Dec 6;12(12):e1006451. doi: 10.1371/journal.pgen.1006451 (PMC5140057; doi:10.1371/journal.pgen.1006451)

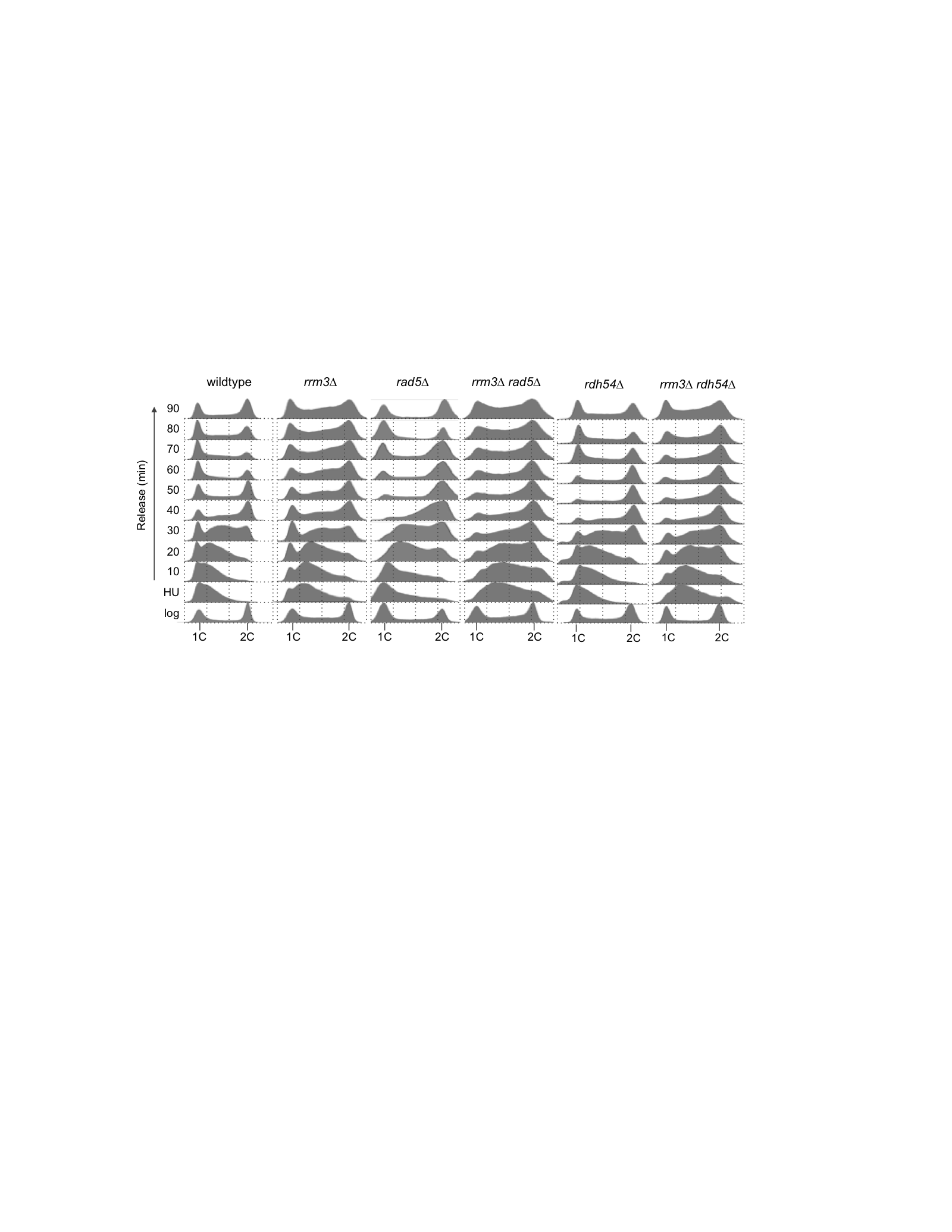

Supplement: S1 Fig — (TIF) [file pgen.1006451.s001.tif]

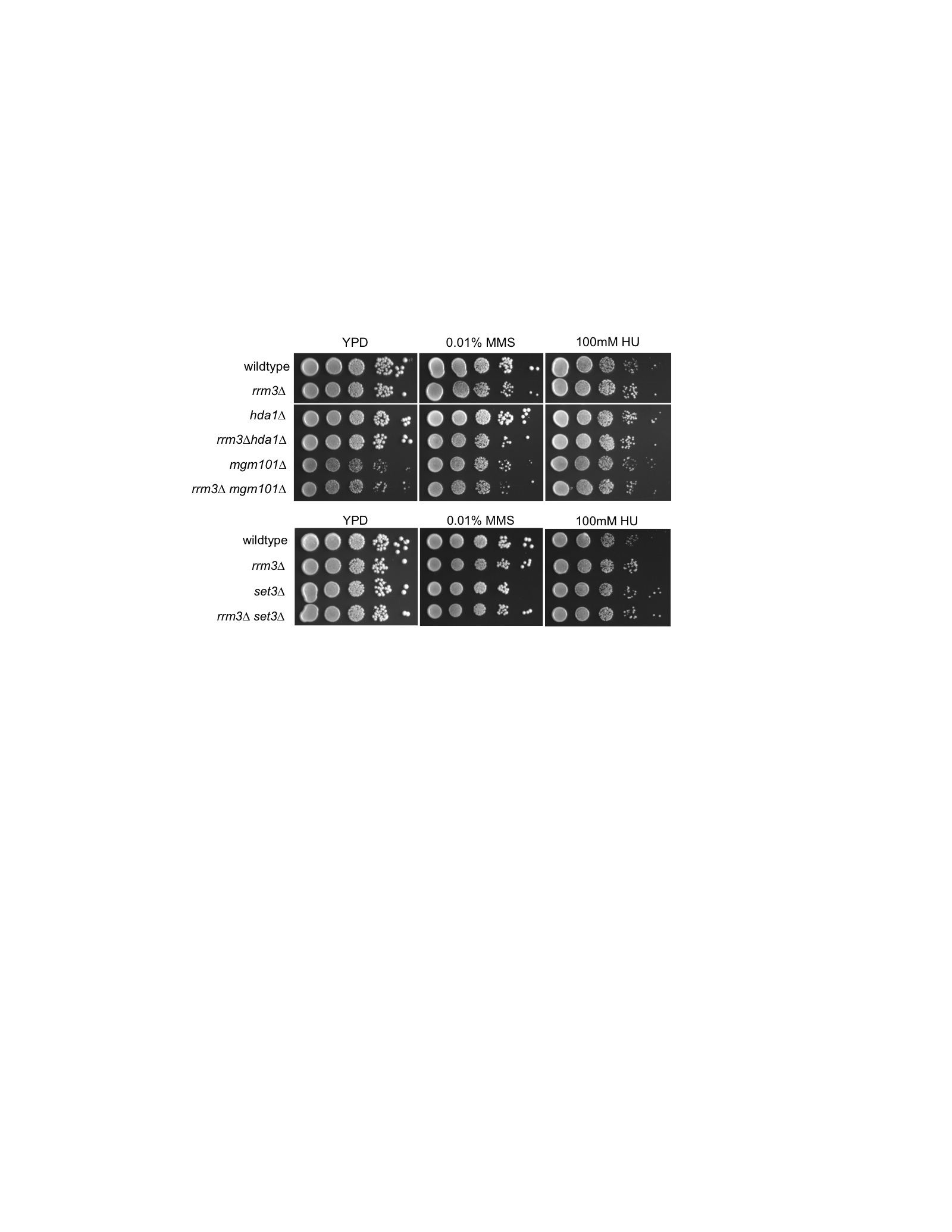

Supplement: S2 Fig — Chromatin association of Hda1 and Set3 significantly decreased in the absence of Rrm3 whereas Mgm101 increased. Deletion of MGM101 resulted in the ‘petite’ phenotype. Serial dilutions of exponentially growing cultures of the indicated mutants were spotted on rich media containing 0.01% MMS or 100 mM HU, or no drug (YPD), followed by incubation for 2–3 days at 30℃. (TIF) [file pgen.1006451.s002.tif]

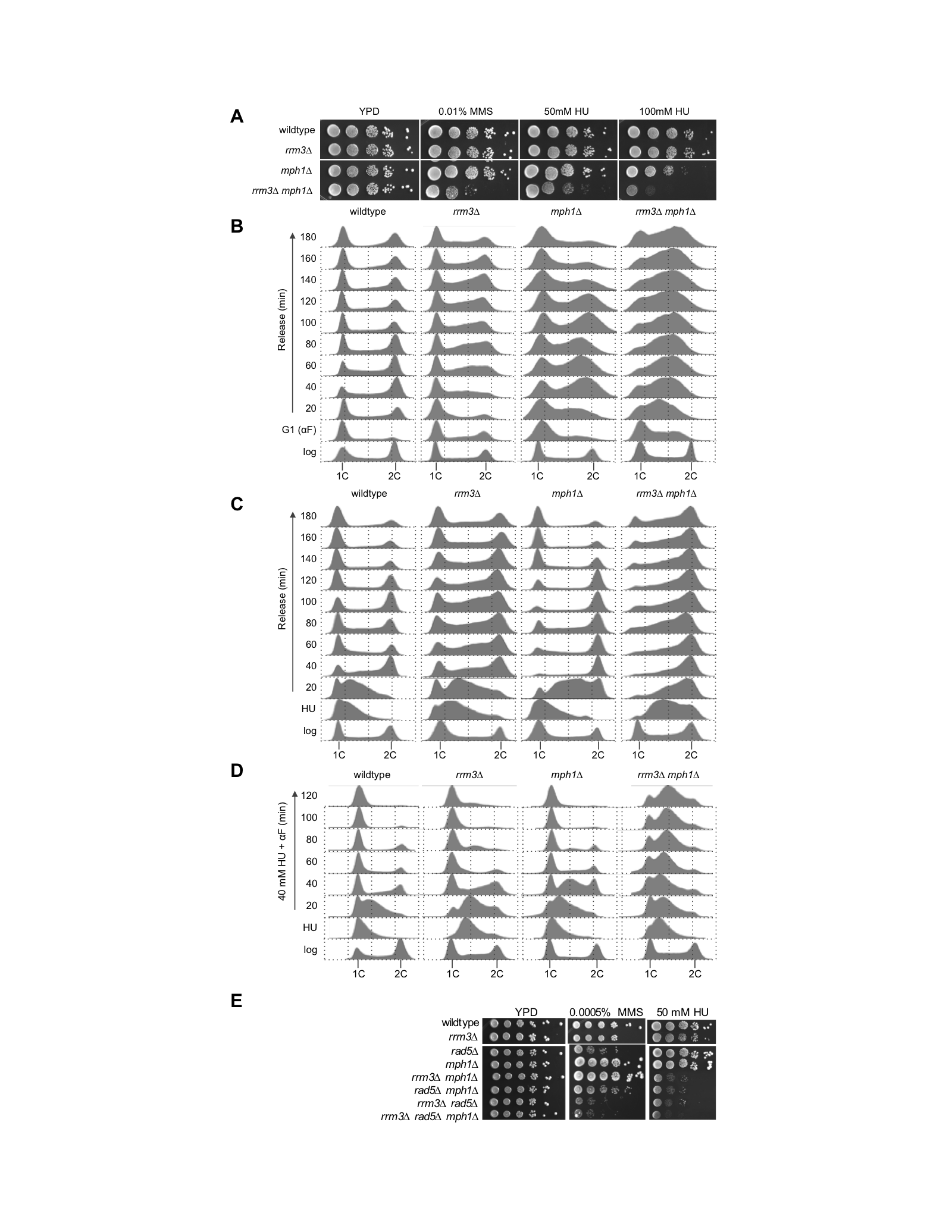

Supplement: S3 Fig — (A) Deletion of RRM3 causes a synergistic increase in HU and MMS sensitivity of cells lacking the DNA helicase Mph1. Absence of Mph1 causes delayed S phase progression of rrm3Δ cells in the absence of HU (B), after release from HU into an undisturbed S phase (C), and, most severely, during chronic exposure to HU (D). (E) Adding the mph1Δ mutation to the rrm3Δ rad5Δ mutations leads to a slight increase in sensitivity to 50 mM HU, but not to 0.0005% MMS. (TIF) [file pgen.1006451.s003.tif]

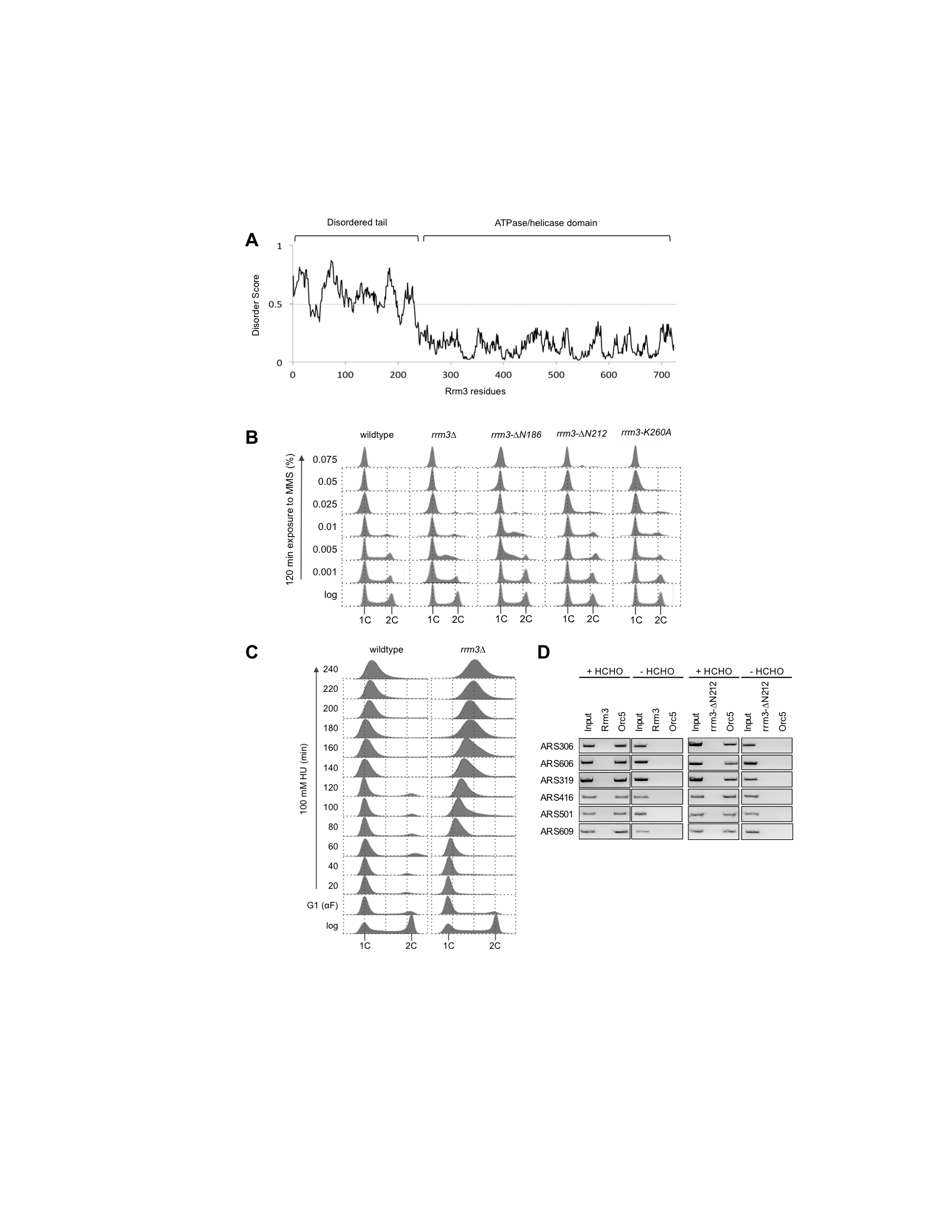

Supplement: S4 Fig — (A) The N-terminal tail of Rrm3 is predicted to be unstructured. A disorder score of >0.5 indicates a disordered residue [88]. (B) In contrast to HU, rrm3-ΔN212 and rrm3Δ mutants arrest with 1C DNA content when exposed to MMS. (C) DNA content analysis of an rrm3Δ mutant released from G1 arrest into 100 mM HU. (D) Rrm3 and rrm3-ΔN212 do not associate with ARS306, ARS319, ARS416, ARS501, ARS606 and ARS609. Association with origins of replication was analyzed by chromatin-immunoprecipitation in cells from asynchronous cultures with or without cross-linking with formaldehyde (HCHO). (TIF) [file pgen.1006451.s004.tif]
