# Supplementary material for: A Novel Rrm3 Function in Restricting DNA Replication via an Orc5-Binding Domain Is Genetically Separable from Rrm3 Function as an ATPase/Helicase in Facilitating Fork Progression
Source: PLoS Genet. 2016 Dec 6;12(12):e1006451. doi: 10.1371/journal.pgen.1006451 (PMC5140057; doi:10.1371/journal.pgen.1006451)
Supplement: S1 Table — (PDF) [file pgen.1006451.s005.pdf]

S1 Table. Proteins that undergo significant changes in cells lacking Rrm3 in the presence and absence of HU

Protein	Fold Change - HU	Description
Rad5	5.1	RING domain-containing ubiquitin ligase involved in error free DNA-damage tolerance pathways
Top2	1.9	Type II topoisomerase that catalyzes decatenation of DNA
Rdh54	1.8	DNA-dependent ATPase with function in homologous recombination
Smc1	1.5	Subunit of the cohesin complex that associates with SMC3 and is required for sister chromatid cohesion
Sif2	1.5	Subunit of Set3C histone deacetylase complex
Mgm101	1.4	Role in mitochondrial DNA recombinational repair and interstrand cross-link repair
Smc3	1.3	Subunit of the cohesin complex that associates with SMC1 required for sister chromatid cohesion
Scc3/Irr1	1.3	Subunit of the cohesin complex, thought to close the cohesion ring
Hda1	-1.7	Putative catalytic subunit of a class II histone deacetylase complex
Mcm4	-1.9	Essential helicase component of heterohexameric MCM2-7 complexes
Rsc1	-2.0	Component of the RSC chromatin remodeling complex

Protein	Fold Change + HU	Description
Rdh54	2.6	DNA-dependent ATPase with function in homologous recombination
Spt7	1.7	SAGA core component that may regulate Spt20 and Ada1, components of the SAGA complex.
Ybp2	1.6	Central kinetochore associated protein
Cdc28	1.6	Associates with six B-type cyclins to direct mitotic spindle assembly in S-phase and spindle function during mitosis
Rpt4	1.6	ATPase of the 19S regulatory particle of the 26S proteasome. One of six ATPases of the regulatory particle, involved in degradation of ubiquitinated substrates
Mgm101	1.5	Role in mitochondrial DNA recombinational repair and interstrand cross-link repair
Mph1	1.4	3'-5' DNA helicase involved in error-free bypass of DNA lesions
Top2	1.4	Type II topoisomerase that catalyzes decatenation of DNA
Rsc9	-1.9	Component of the RSC chromatin remodeling complex which targets genes regulated by stress
Set3	-2.7	Component of the 7-subunit HDAC complex with a role in transcription
Vps72	-2.7	Part of the SWR1 complex, exchanges histone variant H2AZ (Htz1p) for chromatin-bound histone H2A
