# Supplementary material for: A Novel Rrm3 Function in Restricting DNA Replication via an Orc5-Binding Domain Is Genetically Separable from Rrm3 Function as an ATPase/Helicase in Facilitating Fork Progression
Source: PLoS Genet. 2016 Dec 6;12(12):e1006451. doi: 10.1371/journal.pgen.1006451 (PMC5140057; doi:10.1371/journal.pgen.1006451)
Supplement: S2 Table — (PDF) [file pgen.1006451.s006.pdf]

S2 TABLE. Yeast Strains used in this study.

Strain ID1	Genotype
EGY48[1]	*MATα ura3-52, trp1Δ63, his3Δ200, LexA*$_{op\ (x6)}$*-LEU2*
KHSY304[2]	*MATα ura3-52, trp1Δ63, his3Δ200, rrm3::TRP1*
KHSY745	*ura3-52/ura3-52, trp1Δ63/ trp1Δ63, his3Δ200/his3Δ200, RRM3/rrm3::TRP1, MRC1/mrc1::HIS3*
KHSY802	*MATa ura3-52, leu2Δ1, trp1Δ63, his3Δ200, lys2ΔBgl, hom3-10, ade2Δ1, ade8, YEL069C::URA3*
KHSY1063	*MATa ura3-52, leu2Δ1, trp1Δ63, his3Δ200, lys2ΔBgl, hom3-10, ade2Δ1, ade8, YEL069C::URA3, rrm3::TRP1*
KHSY1065	*ura3-52/ura3-52, leu2Δ1/leu2Δ1, trp1Δ63/trp1Δ63, his3Δ200/his3Δ200, lys2ΔBgl/lys2ΔBgl, hom3-10/hom3-10, ade2Δ1/ade2Δ1, ade8/ade8. YEL069C::URA3/YEL069C::URA3, RAD53/rad53::his3, SML1/sml1::G418, RRM3/rrm3::TRP1*
KHSY1133	*ura3-52, lys2-801 amber, ade2-101 ochre, trp1-Δ63, his3-Δ200, leu2-Δ1, rad9::G418, rrm3::URA3*
KHSY1157	*ura3-52/ura3-52, leu2Δ1/leu2Δ1, trp1Δ63/trp1Δ63, his3Δ200/his3Δ200, lys2ΔBgl/lys2ΔBgl, hom3-10/hom3-10, ade2Δ1/ade2Δ1, ade8/ade8. YEL069C::URA3/YEL069C::URA3*
KHSY1557	*MATa ura3-52, leu2Δ1, trp1Δ63, his3Δ200, lys2ΔBgl, hom3-10, ade2Δ1, ade8, YEL069C::URA3, mph1::HIS3*
KHSY1713	*MATa ura3-52, leu2Δ1, trp1Δ63, his3Δ200, lys2ΔBgl, hom3-10, ade2Δ1, ade8, YEL069C::URA3, rrm3::TRP1, mph1::HIS3*
KHSY2331	*MATa ura3-52, leu2Δ1, trp1Δ63, his3Δ200, lys2ΔBgl, hom3-10, ade2Δ1, ade8, YEL069C::URA3, lig4::loxP-G418-loxP*
KHSY4570	*MATa ura3-52, trp1Δ63, his3Δ200*
KHSY4743	*ura3-52/ura3-52, trp1Δ63/ trp1Δ63, his3Δ200/his3Δ200, RRM3/rrm3::TRP1*
KHSY5143	*MATα ura3-52, trp1Δ63, his3Δ200, arg4::G418, lys2::HIS3*
KHSY5144	*MATα ura3-52, trp1Δ63, his3Δ200, arg4::G418, lys2::HIS3, rrm3::TRP1*
KHSY5145	*MATa ura3-52, leu2Δ1, trp1Δ63, his3Δ200, lys2ΔBgl, hom3-10, ade2Δ1, ade8, YEL069C::URA3, rad5::HIS3*
KHSY5146	*MATa ura3-52, leu2Δ1, trp1Δ63, his3Δ200, lys2ΔBgl, hom3-10, ade2Δ1, ade8, YEL069C::URA3, rrm3::TRP1, rad5::HIS3*
KHSY5147	*MATa ura3-52, leu2Δ1, trp1Δ63, his3Δ200, lys2ΔBgl, hom3-10, ade2Δ1, ade8, YEL069C::URA3, rdh54::HIS3*
KHSY5148	*MATa ura3-52, leu2Δ1, trp1Δ63, his3Δ200, lys2ΔBgl, hom3-10, ade2Δ1, ade8, YEL069C::URA3, rrm3::TRP1, rdh54::HIS3*
KHSY5149	*MATa ura3-52, leu2Δ1, trp1Δ63, his3Δ200, lys2ΔBgl, hom3-10, ade2Δ1, ade8, YEL069C::URA3, rad5::HIS3 rdh54::G418*

KHSY5150	*MATa ura3-52, leu2Δ1, trp1Δ63, his3Δ200, lys2ΔBgl, hom3-10, ade2Δ1, ade8, YEL069C::URA3, rrm3::TRP1, rdh54::HIS3, rad5::TRP1*
KHSY5151	*MATa ura3-52, leu2Δ1, trp1Δ63, his3Δ200, lys2ΔBgl, hom3-10, ade2Δ1, ade8, YEL069C::URA3, set3::HIS3*
KHSY5152	*MATa ura3-52, leu2Δ1, trp1Δ63, his3Δ200, lys2ΔBgl, hom3-10, ade2Δ1, ade8, YEL069C::URA3, rrm3::TRP1, set3::HIS3*
KHSY5153	*MATa ura3-52, leu2Δ1, trp1Δ63, his3Δ200, lys2ΔBgl, hom3-10, ade2Δ1, ade8, YEL069C::URA3, tof1::HIS3*
KHSY5154	*MATa ura3-52, leu2Δ1, trp1Δ63, his3Δ200, lys2ΔBgl, hom3-10, ade2Δ1, ade8, YEL069C::URA3, rrm3::TRP1, tof1::HIS3*
KHSY5155	*MATa ura3-52, leu2Δ1, trp1Δ63, his3Δ200, lys2ΔBgl, hom3-10, ade2Δ1, ade8, YEL069C::URA3, ies4::HIS3*
KHSY5156	*MATa ura3-52, leu2Δ1, trp1Δ63, his3Δ200, lys2ΔBgl, hom3-10, ade2Δ1, ade8, YEL069C::URA3, rrm3::TRP1, ies4::HIS3*
KHSY5157	*MATa ura3-52, leu2Δ1, trp1Δ63, his3Δ200, lys2ΔBgl, hom3-10, ade2Δ1, ade8, YEL069C::URA3, hda1::HIS3*
KHSY5158	*MATa ura3-52, leu2Δ1, trp1Δ63, his3Δ200, lys2ΔBgl, hom3-10, ade2Δ1, ade8, YEL069C::URA3, rrm3::TRP1, hda1::HIS3*
KHSY5159	*MATa ura3-52, leu2Δ1, trp1Δ63, his3Δ200, lys2ΔBgl, hom3-10, ade2Δ1, ade8, YEL069C::URA3, mgm101::HIS3*
KHSY5160	*MATa ura3-52, leu2Δ1, trp1Δ63, his3Δ200, lys2ΔBgl, hom3-10, ade2Δ1, ade8, YEL069C::URA3, rrm3::TRP1, mgm101::HIS3*
KHSY5161	*MATa ura3-52, leu2Δ1, trp1Δ63, his3Δ200, lys2ΔBgl, hom3-10, ade2Δ1, ade8, YEL069C::URA3, sml1::G418*
KHSY5162	*MATa ura3-52, leu2Δ1, trp1Δ63, his3Δ200, lys2ΔBgl, hom3-10, ade2Δ1, ade8, YEL069C::URA3, sml1::G418, rrm3::TRP1*
KHSY5163	*MATa ura3-52, leu2Δ1, trp1Δ63, his3Δ200, lys2ΔBgl, hom3-10, ade2Δ1, ade8, YEL069C::URA3, sml1::G418, rrm3::TRP1, rad53::HIS3*
KHSY5164	*ura3-52/ura3-52, leu2Δ1/leu2Δ1, trp1Δ63/trp1Δ63, his3Δ200/his3Δ200, lys2ΔBgl/lys2ΔBgl, hom3-10/hom3-10, ade2Δ1/ade2Δ1, ade8/ade8. YEL069C::URA3/YEL069C::URA3, rdh54::HIS3/rdh54::HIS3*
KHSY5165	*ura3-52/ura3-52, leu2Δ1/leu2Δ1, trp1Δ63/trp1Δ63, his3Δ200/his3Δ200, lys2ΔBgl/lys2ΔBgl, hom3-10/hom3-10, ade2Δ1/ade2Δ1, ade8/ade8. YEL069C::URA3/YEL069C::URA3, rrm3::TRP1/rrm3::TRP1, rdh54::HIS3/rdh54::HIS3*
KHSY5166	*ura3-52/ura3-52, leu2Δ1/leu2Δ1, trp1Δ63/trp1Δ63, his3Δ200/his3Δ200, lys2ΔBgl/lys2ΔBgl, hom3-10/hom3-10, ade2Δ1/ade2Δ1, ade8/ade8. YEL069C::URA3/YEL069C::URA3, rrm3::TRP1/rrm3::TRP1*
KHSY5167	*ura3-52/ura3-52, leu2Δ1/leu2Δ1, trp1Δ63/trp1Δ63, his3Δ200/his3Δ200, lys2ΔBgl/lys2ΔBgl, hom3-10/hom3-10, ade2Δ1/ade2Δ1, ade8/ade8. YEL069C::URA3/YEL069C::URA3, RRM3/rrm3::TRP1, RDH54/rdh54::HIS3*
KHSY5168	*ura3-52/ura3-52, leu2Δ1/leu2Δ1, trp1Δ63/trp1Δ63, his3Δ200/his3Δ200, lys2ΔBgl/lys2ΔBgl, hom3-10/hom3-10, ade2Δ1/ade2Δ1, ade8/ade8. YEL069C::URA3/YEL069C::URA3, RRM3/rrm3::TRP1*

KHSY5169	*ura3-52/ura3-52, leu2Δ1/leu2Δ1, trp1Δ63/trp1Δ63, his3Δ200/his3Δ200, lys2ΔBgl/lys2ΔBgl, hom3-10/hom3-10, ade2Δ1/ade2Δ1, ade8/ade8. YEL069C::URA3/YEL069C::URA3, RDH54/rdh54::HIS3*
KHSY5170	*ura3-52/ura3-52, leu2Δ1/leu2Δ1, trp1Δ63/trp1Δ63, his3Δ200/his3Δ200, lys2ΔBgl/lys2ΔBgl, hom3-10/hom3-10, ade2Δ1/ade2Δ1, ade8/ade8. YEL069C::URA3/YEL069C::URA3, RRM3/rrm3::TRP1, rdh54::HIS3/rdh54::HIS3*
KHSY5171	*ura3-52/ura3-52, leu2Δ1/leu2Δ1, trp1Δ63/trp1Δ63, his3Δ200/his3Δ200, lys2ΔBgl/lys2ΔBgl, hom3-10/hom3-10, ade2Δ1/ade2Δ1, ade8/ade8. YEL069C::URA3/YEL069C::URA3, rrm3::TRP1/rrm3::TRP1, RDH54/rdh54::HIS3*
KHSY5173	*MATa ura3-52, leu2Δ1, trp1Δ63, his3Δ200, lys2ΔBgl, hom3-10, ade2Δ1, ade8, YEL069C::URA3, rrm3::TRP1, mph1::HIS3, rad5::LEU2*
KHSY5177	*MATa ura3-52, leu2Δ1, trp1Δ63, his3Δ200, lys2ΔBgl, hom3-10, ade2Δ1, ade8, YEL069C::URA3, mph1::HIS3, rad5::LEU2*
KHSY5180	*MATa ura3-52, leu2Δ1, trp1Δ63, his3Δ200, lys2ΔBgl, hom3-10, ade2Δ1, ade8, YEL069C::URA3, rad54::HIS3*
KHSY5183	*MATa ura3-52, leu2Δ1, trp1Δ63, his3Δ200, lys2ΔBgl, hom3-10, ade2Δ1, ade8, YEL069C::URA3, rad54::HIS3, rrm3::TRP1*
KHSY5186	*MATa ura3-52, leu2Δ1, trp1Δ63, his3Δ200, lys2ΔBgl, hom3-10, ade2Δ1, ade8, YEL069C::URA3, rad51::HIS3*
KHSY5189	*MATa ura3-52, leu2Δ1, trp1Δ63, his3Δ200, lys2ΔBgl, hom3-10, ade2Δ1, ade8, YEL069C::URA3, rad51::HIS3, rrm3::TRP1*
KHSY5192	*MATa ura3-52, leu2Δ1, trp1Δ63, his3Δ200, lys2ΔBgl, hom3-10, ade2Δ1, ade8, YEL069C::URA3, Rrm3.myc.HIS3*
KHSY5193	*MATa ura3-52, leu2Δ1, trp1Δ63, his3Δ200, lys2ΔBgl, hom3-10, ade2Δ1, ade8, YEL069C::URA3, rrm3-ΔN186.myc.HIS3*
KHSY5194	*MATa ura3-52, leu2Δ1, trp1Δ63, his3Δ200, lys2ΔBgl, hom3-10, ade2Δ1, ade8, YEL069C::URA3, rrm3-ΔN212.myc.HIS3*
KHSY5195	*MATa ura3-52, leu2Δ1, trp1Δ63, his3Δ200, lys2ΔBgl, hom3-10, ade2Δ1, ade8, YEL069C::URA3, rrm3-ΔK260A.myc.HIS3*
KHSY5196	*MATa ura3-52, leu2Δ1, trp1Δ63, his3Δ200, lys2ΔBgl, hom3-10, ade2Δ1, ade8, YEL069C::URA3, rrm3-ΔK260D.myc.HIS3*
KHSY5197	*MATa ura3-52, leu2Δ1, trp1Δ63, his3Δ200, Rrm3.myc.HIS3, Orc5.V5.6xHIS*
KHSY5198	*MATa ura3-52, leu2Δ1, trp1Δ63, his3Δ200, rrm3-ΔN186.myc.HIS3, Orc5.V5.6xHIS*
KHSY5199	*MATa ura3-52, leu2Δ1, trp1Δ63, his3Δ200, rrm3-ΔN212.myc.HIS3, Orc5.V5.6xHIS*

[1] From Gyuris *et al.* (1993) Cell **75**(4): p. 791-803.
[2] All KHSY strains are derived from the S288C background.
