# Supplementary material for: A Novel Rrm3 Function in Restricting DNA Replication via an Orc5-Binding Domain Is Genetically Separable from Rrm3 Function as an ATPase/Helicase in Facilitating Fork Progression
Source: PLoS Genet. 2016 Dec 6;12(12):e1006451. doi: 10.1371/journal.pgen.1006451 (PMC5140057; doi:10.1371/journal.pgen.1006451)
Supplement: S3 Table — (PDF) [file pgen.1006451.s007.pdf]

S3 Table. Plasmids used in this study

Plasmid	Description	Reference
pRS315	*CEN/ARS, LEU2*	(Sikorski and Hieter, 1989)
pEG202	LexA bait plasmid*, HIS3,* 2µm	(Estojak et al., 1995)
pJG4-5*	*GAL1* promoter-$GAL4_{AD}$, *TRP1,* 2µm	(Schmidt et al., 2002)
pR5-28	*CEN/ARS, LEU2, RAD5*	(Pages et al., 2008)
pR5-19	*CEN/ARS, LEU2, rad5-C914A/C917A*	(Pages et al., 2008)
pKHS124	*pJG-4-5*-RRM3*	(Schmidt et al., 2002)
pKHS126	*pEG202-RRM3*	(Schmidt et al., 2002)
pKHS133	*pEG202-rrm3-ΔN54*	(Schmidt et al., 2002)
pKHS135	*pEG202-rrm3ΔN230*	(Schmidt et al., 2002)
pKHS136	*pRS315-rrm3-ΔN54*	this study
pKHS137	*pRS315-rrm3-ΔN230*	this study
pKHS170	*pRS315-RRM3*	this study
pKHS174	*pRS315-rrm3-F41A/F42A*	this study
pKHS183	*pEG202-rrm3-F41A/F42A*	(Schmidt et al., 2002)
pKHS645	*pRS315-rrm3-NΔ142*	this study
pKHS646	*pRS315-rrm3-ΔN186*	this study
pKHS647	*pRS315-rrm3-Δ212*	this study
pKHS648	*pRS315-rrm3-K260A*	this study
pKHS649	*pRS315-rrm3-K260D*	this study
pKHS653	*peg202-rrm3-ΔN186*	this study
pKHS654	*pEG202-rrm3-ΔN212*	this study
pKHS655	*pEG202-rrm3-ΔN230*	(Schmidt et al., 2002)
pKHS656	*pJG4-5*-ORC5*	this study
pKHS736	*CEN/ARS,LEU2, rad5-Q1106D*	this study

Estojak, J., Brent, R., and Golemis, E.A. (1995). Correlation of two-hybrid affinity data with in vitro measurements. Mol Cell Biol *15*, 5820-5829.

Pages, V., Bresson, A., Acharya, N., Prakash, S., Fuchs, R.P., and Prakash, L. (2008). Requirement of Rad5 for DNA polymerase zeta-dependent translesion synthesis in Saccharomyces cerevisiae. Genetics *180*, 73-82.

Schmidt, K.H., Derry, K.L., and Kolodner, R.D. (2002). Saccharomyces cerevisiae RRM3, a 5' to 3' DNA helicase, physically interacts with proliferating cell nuclear antigen. J Biol Chem *277*, 45331-45337.

Sikorski, R.S., and Hieter, P. (1989). A system of shuttle vectors and yeast host strains designed for efficient manipulation of DNA in Saccharomyces cerevisiae. Genetics *122*, 19-27.
